# Click chemistry approaches for developing carbonic anhydrase inhibitors and their applications

**DOI:** 10.1080/14756366.2023.2166503

**Published:** 2023-01-13

**Authors:** Andrea Angeli, Claudiu T. Supuran

**Affiliations:** Department of NEUROFARBA, Section of Pharmaceutical and Nutraceutical Sciences, University of Florence, Florence, Italy

**Keywords:** Carbonic anhydrase, click chemistry, sulphonamide, coumarin, carbonic anhydrase inhibitors

## Abstract

Click chemistry reactions constitute an important and relatively new approach in the medicinal chemistry toolbox and offer substantial advantages to medicinal chemists in terms of overcoming the limitations of facile chemical synthesis, increased throughput, yields and improved quality of compound libraries. Over the last two decades, click chemistry has been widely used in different aspects of carbonic anhydrase (CA, EC 4.2.1.1) modulators drug design. This review provides an overview of the general principles and applications of click chemistry in CA-related research, including the design of selective CA inhibitors (CAIs), their applications against several diseases such as innovative anticancer and anti-infective agents, fluorescent and radiopharmaceutical probes as new theranostic agents, as well as their application in protein labelling.

## Introduction

The concept of click chemistry was introduced concomitantly by Sharpless and Meldal who used the well-known cycloaddition reaction to couple a wide range of organic scaffolds, which soon thereafter received massive attention in several research fields, including organic chemistry, material sciences as well as drug design and discovery^1–4^. Click chemistry is referred to as a set of powerful, highly reliable and stereoselective reactions, which generate substances quickly and reliably by joining small units together through heteroatom linkages (C-X-C). Indeed, there are several stringent criteria that a reaction must satisfy to be useful in this context such as to be “modular, wide in scope, give very high yields, generate only inoffensive byproducts that can be removed by non-chromatographic methods, and be stereospecific”^1^. There are a number of reactions that fulfil these criteria mentioned above, such as cycloaddition of alkynes and azides, nucleophilic ring-opening reactions (such as thiol–epoxy reaction, aziridines and aziridinium ions opening reactions, etc.), inverse electron-demand Diels-Alder (IEDDA) reactions, thiol-ene reactions and sulfur(VI) fluoride exchange (SuFEx) reactions. Huisgen’s 1,3-dipolar cycloaddition of alkynes and azides has been used as the frontrunner, especially when used with copper-catalysts such as CuAAC (Cu(I)-catalyzed Azide-Alkyne Click chemistry reaction) as an efficient synthetic tool for the generation of compound libraries to be employed in oncologic, anti-infectives, antivirals and other biomedical research fields^5^. However, the cytotoxic effect of the copper-based catalysts restricts the *in vitro* or *in vivo* applications, with the nowadays tendency to move towards reaction of alkynes using ring-strain which enables an azide–alkyne reaction without the need (cyto)toxic copper catalysts^6^. The 1,2,3-triazole ring present in pharmacologically active molecules, is not a mere passive linker, as this functionality shows several favourable physicochemical properties such as large dipole moment, π-π interaction with aromatic rings (e.g. phenyl rings), hydrogen bonding with hydrogen bond donors or coordination with metal ions, but also good water solubility and high stability under physiological conditions^7^. Due to these features, the 1,2,3-triazole ring is an interesting bioisostere of the amide moiety, showing good stability to hydrolysis, in contrast to the amide bond which is susceptible to nucleophilic attack from enzymes and other nucleophilic species. As mentioned above, the 1,2,3-triazole-based molecules obtained by click chemistry exhibit a broad spectrum of biological properties and have been used as leads for the development of anticancer, antibacterial, antiviral, and biomedical imaging agents^1–5^.

The ubiquitous enzyme superfamily of carbonic anhydrases (CAs, EC 4.2.1.1) plays a pivotal role in most organisms/tissues, being involved in a multitude of physiologic and pathologic functions, due to the catalysis of a simple but essential reaction, the hydration of CO_2_ to bicarbonate and protons^8–11^. To date, eight genetically distinct CA families (α-, β-, γ-, δ-, ζ-, η-, θ- and ι-class) are known and they are expressed in organisms all over the phylogenetic tree, making their modulation a target for a diversity of diseases^12^^,^^13^. In particular, CA inhibitors (CAIs) found clinical relevance as diuretics, antiglaucoma drugs, antiepileptics, antiobesity and antitumor agents and, in the last decades, they also emerged as useful tools in diseases usually not associated with this class of pharmacological agents such as neuropathic pain, cerebral ischaemia, oxidative stress, rheumatoid arthritis, Alzheimer’s disease and as anti-infective agents^10^^,^^14–16^. The rather unusual usefulness of CAIs in so many diverse biomedical applications is probably due to the presence of 15 different human isoforms which differ by their catalytic activity, subcellular localisation, physiological functions and involvement in different pathologies^17^^,^^18^. However, the conserved active site architecture among the human isoforms has made the development of isoform selective CAIs a rather difficult challenge for medicinal chemists. The increased knowledge of CA structural biology/biochemistry over last decades, allowed the observation that amino acid residues at the rim of active site are among the most variable ones between the various CA isoforms, leading to the concept of the “tail-approach” for the design of isoform-selective inhibitors^19^. This approach consists in appending one or more ‘tails’ to a scaffold incorporating a zinc binding group (ZBG) such as sulphonamide or its bioisosteres, leading to an elongated molecule which will be able to interact with the amino acid residues in the middle and outer rims of the active site cavity, which, as mentioned above, are the regions with the highest variability among the different isoforms^19^. This approach led to the development of potent and selective CAIs, also by using the click chemistry methodologies mentioned above, which provided an easy way to synthesise large libraries of isoform-selective compounds. The purpose of this review is to highlight the most significant contributions in the development of CAIs by using click chemistry, as well as their application in drug discovery, imaging, and diagnostics over the past years.

## Click tailing to obtain isoform-selective CAIs

**Figure 1.**
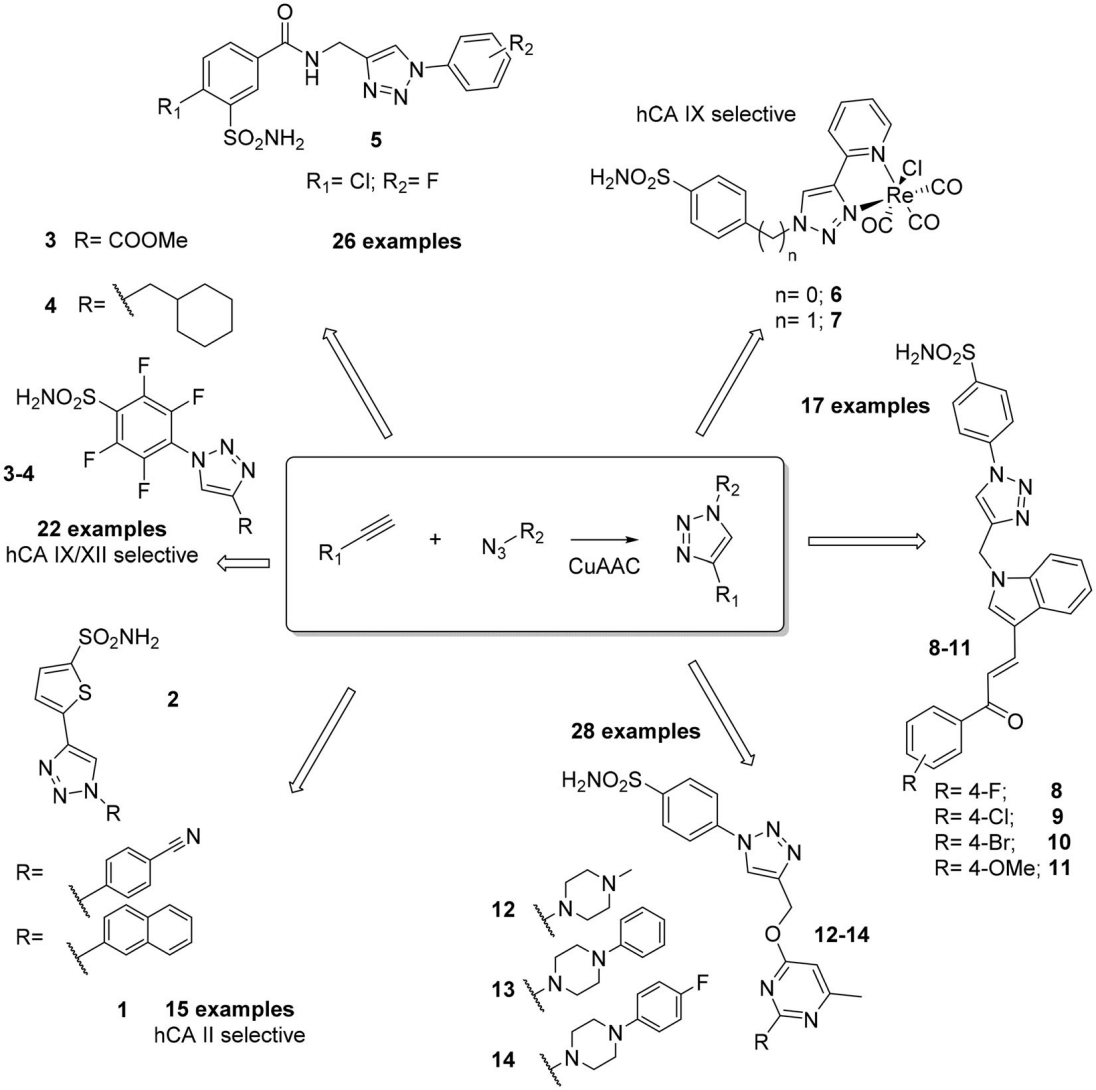
Structures of selected sulphonamide CAIs **1–14** developed by using the click-tailing strategy in the last 10 years.

**Figure 2.**
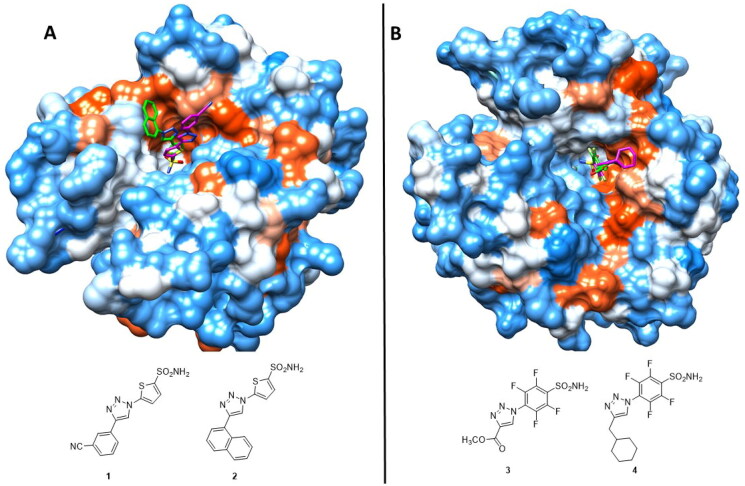
(**A)** Superimposition of sulphonamides **1** (magenta, PDB: 4BF6) and **2** (green, PDB: 4BF1) in complex with hCA II, (**B)** Superimposition of compounds **3** (green, PDB: 4DZ7) and **4** (magenta, PDB: 4DZ9) in complex with hCA II. Hydrophobic (red) and hydrophilic (blue) surfaces of the enzyme/active site cavity are highlighted.

One of the first contribution in using click chemistry for obtaining was reported by Supuran’s group^20^, who synthesised a series of 2-thiophene-sulphonamides incorporating 1-substituted aryl-1,2,3-triazolyl moieties (Figure 1) by using CuAAC as catalyst, and investigated their affinity against the cytosolic isoforms hCA I, II and the transmembrane ones hCA IX and XII. All compounds showed high affinity for hCA II in the low nanomolar range (of 2.2–7.7 nM)^20^. On the other hand, these compound were observed to be weak inhibitors of hCA I and, and of the tumour-associated hCA IX and XII, exhibiting an affinity ranging between 3.4 to 811 nM. In addition, the X-ray crystal structures of two adducts complexed to hCA II (**1,2**) were reported, evidencing the reasons of the high affinity for this enzyme and, in particular, the importance of the tail which adopted two very different orientations when bound to the active site (Figure 2)^20^.

Subsequently, the same research group employed the click-tailing approach for the synthesis of two homologous series of 4-(R-1H-1,2,3-triazol-1- yl)-benzenesulfonamides and 2,3,5,6-tetrafluoro-4–(5-R-1H-1,2,3-triazol-1-il)benzenesulfonamide incorporating a large variety of different moieties (**3,4**, Figure 2) and investigated the inhibition of the same four human isoforms, hCA I, II, IX and XII^21^. The tails chosen incorporated various chemical functionalities, such as aromatic, aliphatic, cycloalkyl, halogeno, hydroxyl or aminoalkyl moieties, in order to achieve a major chemical diversity, which influenced the inhibition profile in terms of selectivity against various isoforms. Indeed, the novel compounds were observed to be selective against the tumour-associated hCA IX and XII isoforms, with inhibition constants in the low nanomolar/subnanomolar range. They showed a medium inhibition potency against the cytosolic hCA I and II- Furthermore, two derivatives (**3**, **4**) were crystallised in complex with hCA II (Figure 2)^21^.

The click tail approach has been used by Arifuddin’s group, who investigated benzenesulfonamides with a 3-sulfamoyl moiety of type **5**, for obtaining a novel series of 1,2,3-triazole derivatives linked through an amide linker to the aryltriazole fragments, and evaluated them against four human isoforms (hCA I, II, IV and IX, Figure 1)^22^. The reported compounds were observed to behave as weak inhibitors of hCA IX, whereas one of the derivatives **5** proved to be a selective hCA II inhibitor. Aimene et al. employed the click tail strategy to obtain two tricarbonyl rhenium(I) conjugates with a pendent 4-substituted benzenesulfonamide fragment (**6**,**7**) which were fully characterised by spectroscopic methods, X-ray crystallography and DFT calculations^23^. These two rhenium complexes were investigated as inhibitors of three human isoforms, the cytosolic hCA I, II and the membrane-associated isoforms IX revealing, a pronounced selectivity against the latter one^23^. Arifuddin’s group also reported the synthesis of novel indolylchalcone linked benzenesulfonamide-1,2,3-triazole hybrids **8–11** (Figure 1)^24^. These derivatives were investigated against four CA isoforms (hCA I, II, IX and XII) and, also in this case, the different substituents in the terminal portion of the scaffold appear to play a key role in the isoform selectivity, although the observed selectivity and the structure activity relationship (SAR) appeared difficult to be rationalised. However, one can note compounds selective for hCA IX (**8**,**9**), hCA I (**10**) and hCA XII (**11**) in these series of derivatives.

Manzoor et al. employed the click tail approach in the design and synthesis of new CAIs bearing substituted pyrimidine rings through a linker incorporated for enhancing the flexibility and achieving more interactions at the rim of CA active site, in order to produce selective CAIs (**12–14**, Figure 1)^25^. This series, as many of the others previously discussed, was tested against the four human isoforms hCA I, II, IX and XII. Also in this case, a moderate activity against hCA I was observed, whereas three compounds (**12–14**) exhibited good selectivity against the tumour-related hCA XII. For this reason, the authors investigated their binding mode by molecular docking studies, showing the benzenesulfonamide moiety to accommodate into the active catalytic pocket towards the zinc ion and the triazole tail displaying a binding towards the rim of the hCA IX and hCA XII active sites^25^.

**Figure 3.**
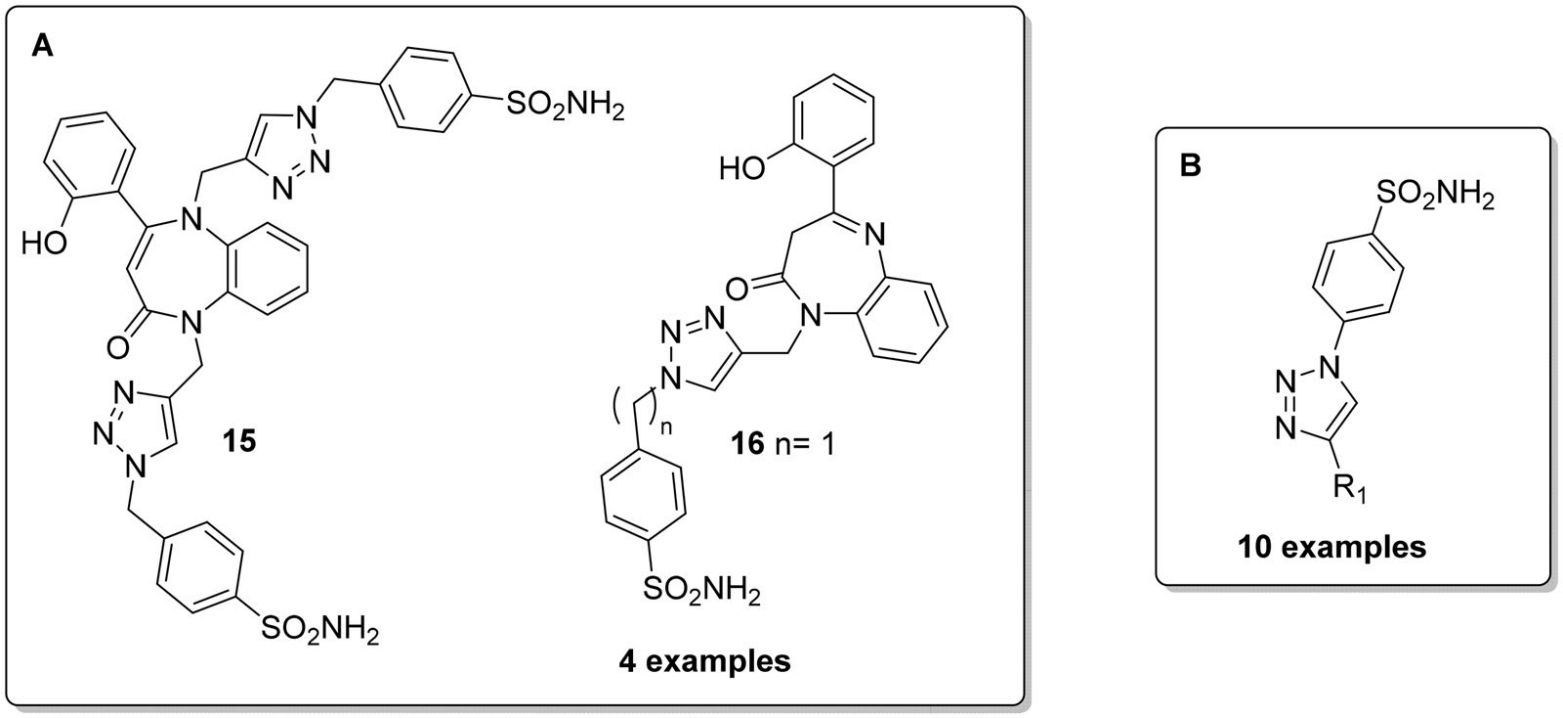
General structure of CAIs reported by Ismail et al. (**A**) and Ewies et al (**B**).

Ismail et al. described the synthesis of novel *N*‐triazolo‐benzene sulphonamides‐1,5‐benzodiazepines **15, 16** (Figure 3(A)) via CuAAC reaction and investigated them against six human isoforms (hCA I, II, IV, VII, IX and XII)^26^. Interestingly, the divalent inhibitor **15** was found to be the most effective inhibitor against these six isoforms compared to the monovalent derivatives **16.** Moreover, derivative **15** showed selectivity against hCA II with a K_I_ of 2.8 nM.

Ewies et al. reported 10 exemples of 1,2,3‐triazol‐1‐yl-benzenesulfonamide derivatives (Figure 3(B)) with different aryl substitutions via CuAAC reaction, with all derivatives showing potent and selective inhibition towards the cytosolic hCA I and II compared to the tumour-associated hCA IX and XII^27^.

**Figure 4.**
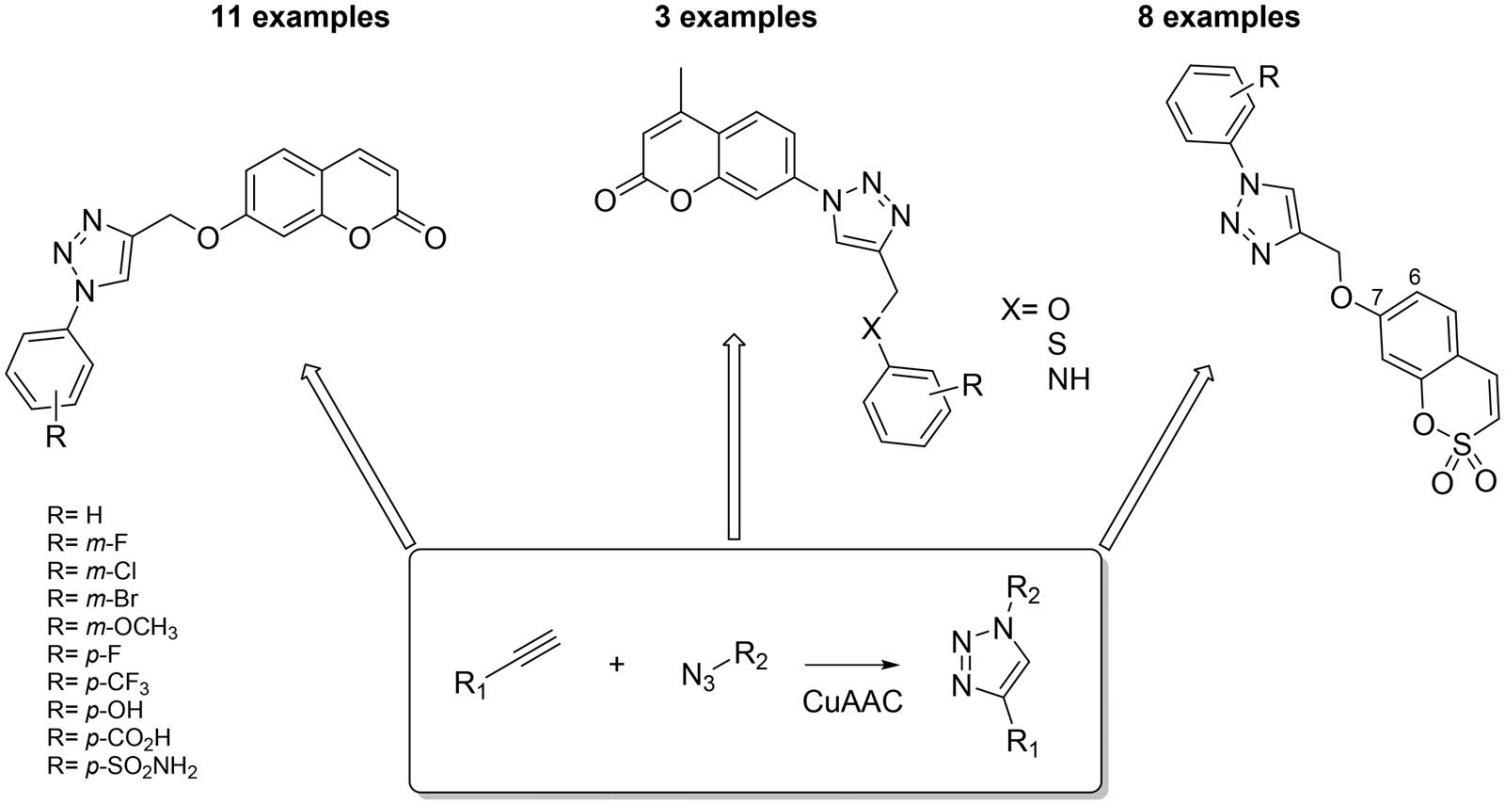
General structures for coumarins and sulfocoumarins-based CAIs developed by click chemistry.

The click tail approach was not employed only to modulate the selectivity of sulphonamide derivatives but, more recently, has been applied to novel chemotypes associated with the inhibition of CAs such as coumarins, a class of compounds that first showed high selectivity mainly for the tumour-associated isoforms hCA IX and XII^28^. This selectivity was attributed to their mechanism of action, since the hydrolysis of the lactone ring generates the active species that thereafter binds the entrance of the CA active site which, as mentioned above, is the most variable portion among the different CA isoforms^29^^,^^30^. Actually, a rather large number of substitution patterns at the coumarin ring have been investigated in order to obtain CAIs with desired physico-chemical and pharmacologic properties. In 2015 the employment of the click chemistry was investigated by Supuran group’ for this class of compounds and, in particular, for obtaining 7-substituted coumarins (Figure 4)^31^. All examples reported so far showed excellent CA IX/XII selective inhibition activity (in the low nanomolar range with K_I_ of 14.3–34.4 nM against hCA IX and of 4.7–37.8 nM against hCA XII) over the cytosolic isoforms hCA I and II.

**Scheme 1.**
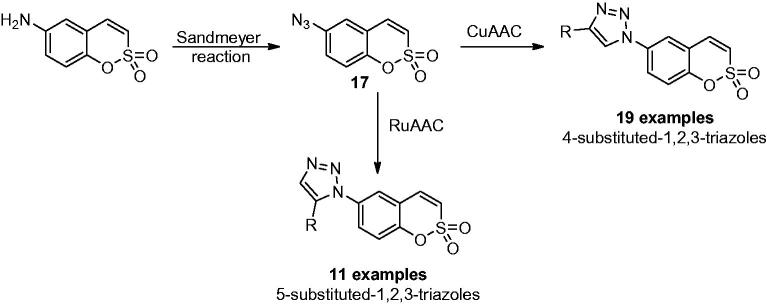
Click-tailing strategies to generate 4-substituted-1,2,3-triazoles and 5-substituted-1,2,3-triazoles sulfocumarins.

Sulfocoumarins (1,2-benzoxathiine 2,2-dioxides) are one of the ultimately discovered prodrug-type class of compounds acting as selective inhibitors against different CA isoforms^32^. They act with a mechanism similar to that of coumarins, being hydrolysed by the sulfatase CA activity to 2-hydroxyphenyl-vinylsulfonic acids, which thereafter bound within the enzyme active site in a region rarely occupied by other classes of inhibitors^33^. In addition, the different substitution pattern on the aromatic ring was shown to be able to influence the potency and selectivity profile of these compounds^33^. For these reasons, many efforts were made to synthesise large libraries of substituted sulfocoumarins, also by employing click chemistry procedures, in order to understand the SAR operating on the isoform selectivity. Zalubovskis’ group employed the classical 1,3-dipolar azide-alkyne cycloaddition click reaction to incorporate chemically diverse moieties in position 6 of sulfocoumarin scaffold^34^^,^^35^. By converting 6-amino sulfocoumarin in the azide derivative **17** by using the Sandmeyer reaction and subsequently reacting it with a series of alkynes, a series of 4-substituted-1,2,3-triazoles were obtained, in the presence of copper-based catalysts, as shown in Scheme 1^34^.

Moreover, the same authors explored the possibility to use ruthenium-based catalysts, instead of copper, to achieve the less explored 5-substituted-1,2,3-triazole sulfocumarins (Scheme 1). Indeed, the use of ruthenium catalyst can facilitate the formation of 1,5-substituted triazoles, although in all cases a mixture of 1,5-(*cis*) and 1,4-(*trans*) triazoles has been reported, which had to be separated, and only in two examples (with 3-F and 3-Cl substituents) the desired product was observed to be formed in superior amount compared to the thermal cycloaddition^35^. This library was assayed against four different hCA isoforms such as the widely expressed hCA I, hCA II and the tumour associate hCA IX and hCA XII, by stopped flow assays^36^. All sulfocoumarins were weak inhibitors of the ubiquitous hCA I and hCA II, with K_i_ in the high micromolar range (up to more than 10 µM). On the other hand, all compounds showed effective inhibition affinity against the tumour-associated isoforms hCA IX and XII, with inhibition constants in the low nanomolar range (K_i_ 7 to 90 nM). In particular, compounds with 5-substituted-1,2,3-triazoles proved to be highly isoform-selective inhibitors, identifying them as suitable drug candidates for clinical trials. The employment of click chemistry on the sulfocoumarin scaffold was continued, in 2015 by Nocentini et al. since substituents at the 7 position were not reported in previous studies (Figure 4)^37^. The authors incorporated a bulky and flexible moiety by means of CuAAC reaction and assayed the compounds against the same human isoforms mentioned above for the 6-substituted sulfocoumarins. The reported series of 7-substituted sulfocoumarins bearing the 1,2,3-triazolyl moieties attached via a CH_2_O linker showed high selectivity against the tumour-associated isoforms hCA IX and XII (K_i_ spanning between 4.5 to 26.8 nM). Interestingly, substituents in position 7 were observed, as in the previous work, to be effective hCA II inhibitors and ineffective hCA IX and XII modulators^38^, proving that the triazole moiety is a key scaffold/linker for influencing the isoform selectivity between the widely expressed hCA II and the tumour-associated isoforms hCA IX and XII.

**Scheme 2.**
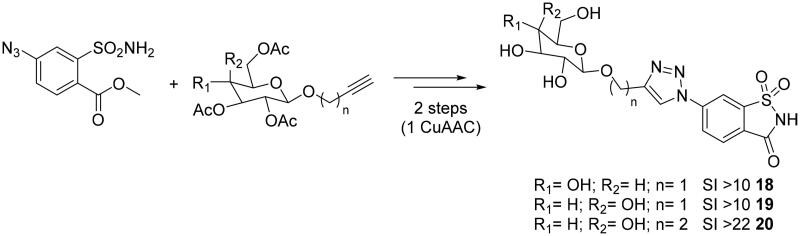
Schematic synthesis for saccharin-glycoconjugates **18–20.**

Considering the opportunity to selectively targeting the extracellular hCA IX and XII *in vivo*, Moeker et al.^39^ and Murray et al.^40^ employed the click tailing approach to append carbohydrate moieties onto the saccharin scaffold, an artificial sweetener, of types **18–20**^39^^,^^40^. In this way, polar triazole glycoconjugates with limited passive membrane permeability and high nanomolar affinity for the tumour-associated hCA IX were obtained^41^^,^^42^. The high selectivity displayed by the saccharin-glycoconjugate reported by Moeker et al. gave the opportunity to focus the attention on modifications affecting the nature of the sugar scaffold, such as the replacement the β-glucose with β-galactose. Cu-catalyzed click reactions were used for introducing an ethyl linker between the glyosidic oxygen and the triazole ring, controlling the binding interactions of the linker and tail region of the saccharin-sugar compound with CA IX active site residues (Scheme 2).

All compounds displayed highly selective inhibition for hCA IX over hCA II, increasing the isoform specificity at least 22-fold for hCA IX (**20**) compared to the classically sulphonamide inhibitor acetazolamide, and proved to be good candidates for being investigated as antitumor agents.

**Scheme 3.**
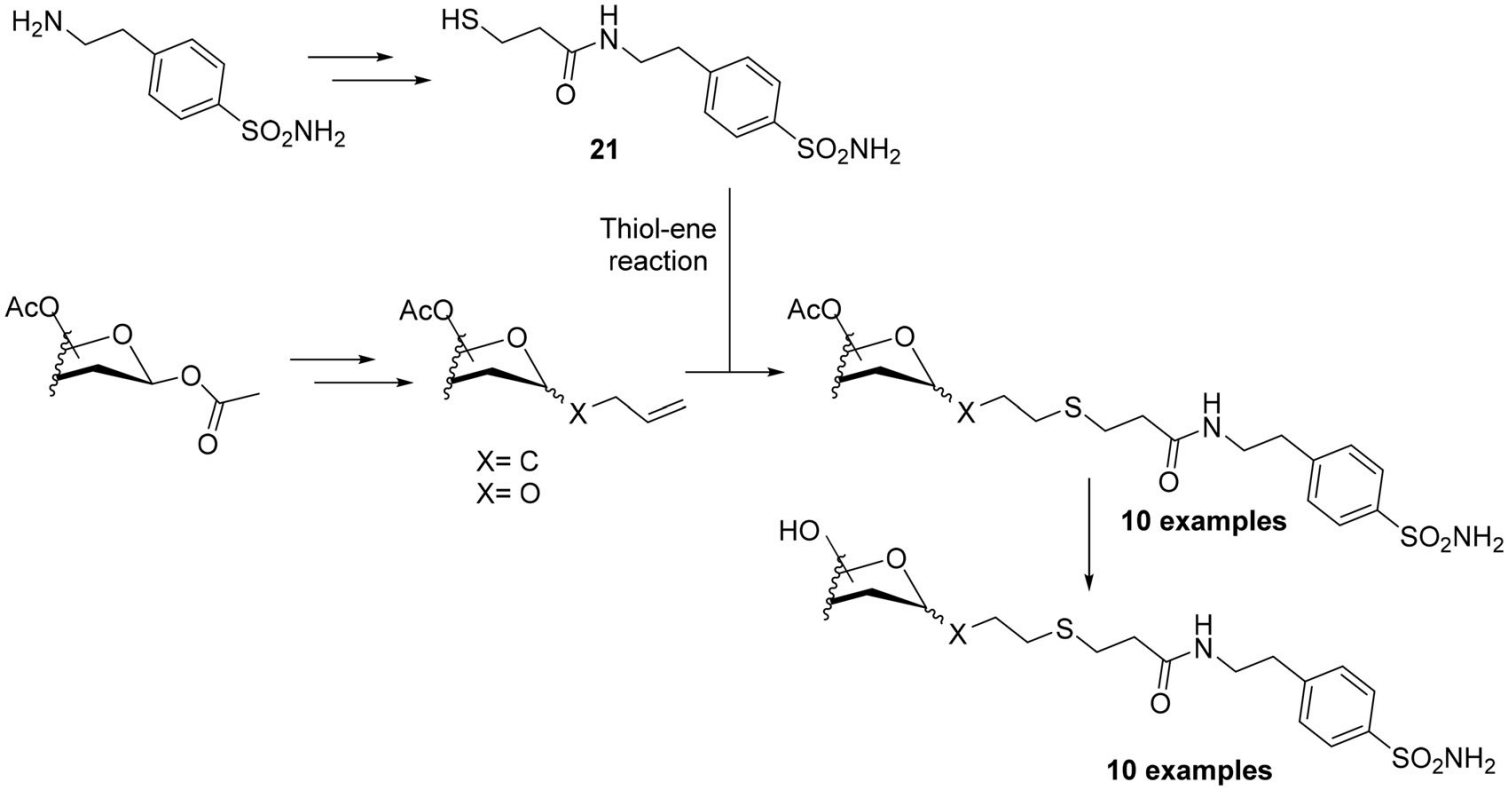
Schematic synthesis of sulphonamide CAIs using the thiol–ene click chemistry reactions.

As seen from the above paragraphs, much of the effort made over the past 10 years in modulating selectivity towards one CA isoform over the others, focussed on a single click reaction, the Huisgen 1,3-dipolar cycloaddition reaction of azides and alkynes. Only Winum’s group employed the thiol–ene click chemistry in 2013 for designing novel sulphonamide CAIs incorporating sugar moieties^43^. As initial step, the authors synthesised the β-mercaptopropionamide of 4–(2-aminoethyl)-benzenesulfonamide (**21**) through a multistep procedure. In the next step, the authors generated five different peracetylated mono or disaccharides (glucopyranose, galactopyranose, mannopyranose, ribofuranose and cellobiose) to which double bonds were appended as the ene component, and reacted them with thiol **21**, to generate the desired thioethers (Scheme 3). All sulphonamides reported in their work, showed excellent selectivity ratio for inhibiting hCA IX over hCA II.

## Click chemistry to develop novel CAIs as anti-infective agents

**Figure 5.**
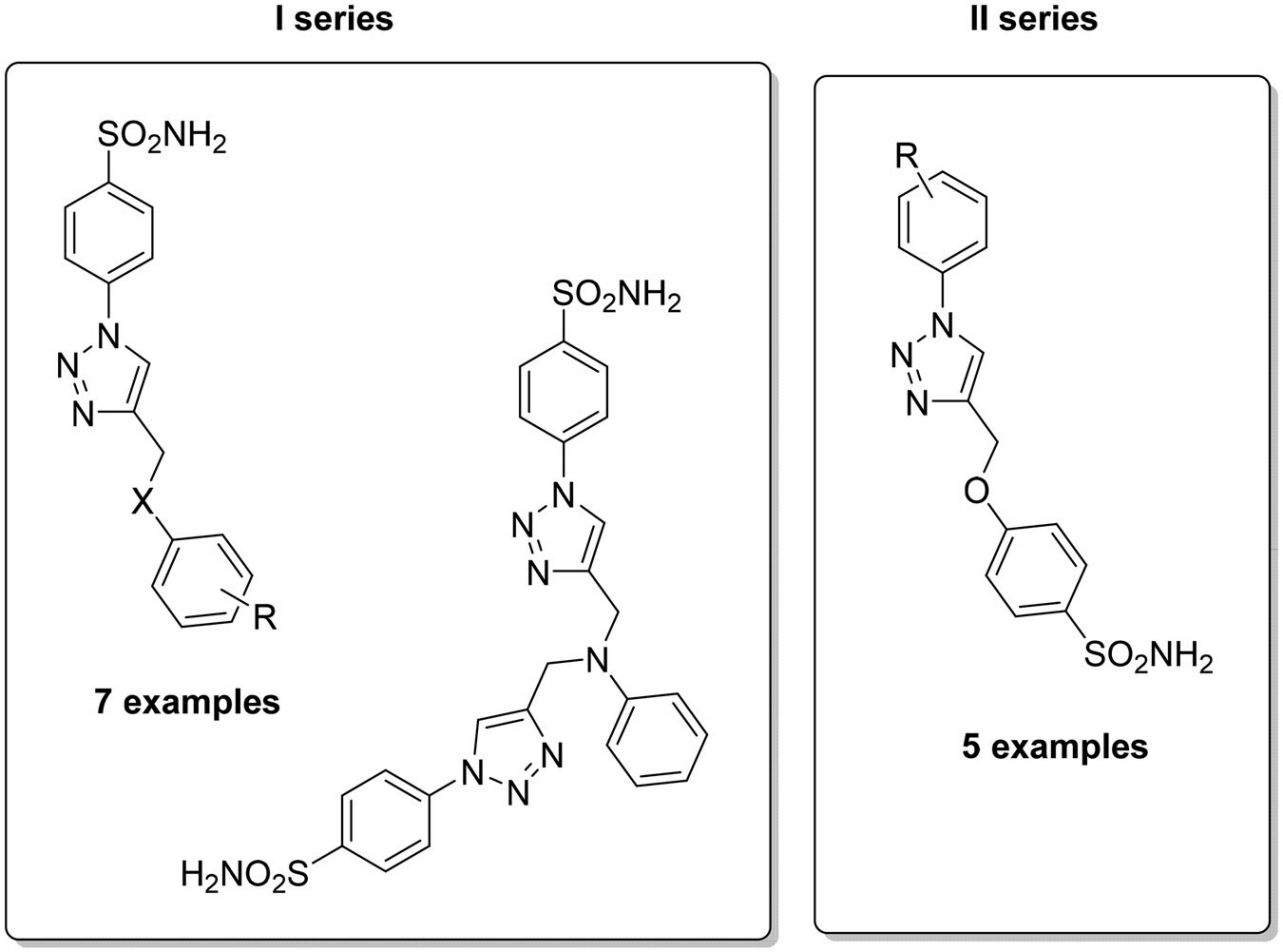
General structures for the development sulphonamide-based CAIs by click chemistry as antiinfective agents.

In recent years, the druggability of CAs present in pathogens as novel anti-infective agents is becoming more and more appealing for design drugs with a novel mechanism of action. Indeed, these enzymes are essential in the life cycle (pH homeostasis and biosynthetic reactions) as well as in the virulence of many bacterial, fungal and protozoan pathogens and, several literature data have shown that their inhibition impairs parasite growth and virulence^44^. In this context, sulphonamide derivatives were studied and screened to obtain such novel anti-infective agents^45^^,^^46^. Supuran’s group developed two different subsets of compounds by using the CuAAC reaction, which differ by the spacer connection among the tail and sulphonamide portion, with the triazole ring being shown to be useful for modulating the inhibition potency trough the well-known tail approach (Figure 5)^47^.

Different bacteria species possessing CAs (*Mycobacterium tuberculosis*, *Vibrio cholerae*) have been included in the study with both α- and β- class CAs being tested. β-mtCA3 from *M. tuberculosis* and two such enzymes (VchCAα and β) from the second one allowed the possibility to discover selective inhibitors, possibly of interest to be investigated for the management of infections provoked by these pathogens. Among them, the first subset of derivatives showed high affinity for VchCAα with inhibition constants in the low nanomolar range (K_I_s between 0.72 and 22.6 nM). On the other hand, compounds of the second subset preferentially inhibited VchCAβ with K_I_s in the range of 54.8–102.4 nM, whereas β-mtCA3 was inhibited with K_I_s in the range of 28.2–192.5 nM^47^.

## Click chemistry as a useful tool for developing hybrid CAIs

**Figure 6.**
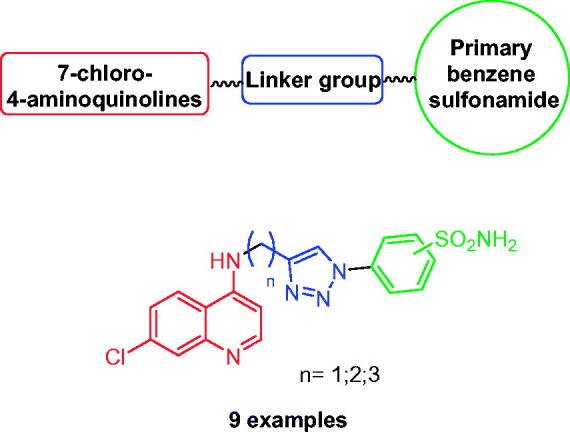
Structure of hybrid CAIs incorporating the 7-chloro-4-aminoquinoline scaffold.

Click chemistry is a useful tool to bioconjugate different pharmacophores in a single scaffold trough a stable linker, such as the 1,2,3-triazole ring. One of the first studies to employ this approach was reported by Poulsen’s group for the development of novel 4-aminoquinolines bearing benzensulfonamide moiety, as potential antimalarial hybrids^48^. Indeed, the inhibition of CA from *Plasmodium falciparum*, the protozoa that causes malaria, with primary sulphonamide showed antimalarial activity^49^. For this reason, the authors merged two antimalarial pharmacophores, the 4-aminoquinolines scaffold and the primary benzene sulphonamide, by using the CuAAC reaction, generating hybrid compounds that showed improved antimalarial activity, stability, and/or solubility compared to the single pharmacophores (Figure 6).

These novel hybrid derivatives showed antimalarial activity *in vitro* both in chloroquine-resistant and chloroquine-sensitive lines, demonstrating the potential to overcome the resistance of chloroquine showed by many *P. falciparum* strains. In addition, the sulphonamide group in position 3 led to a better antimalarial activity compared the analogues with the sulphonamide group in position 4 of the aromatic ring^48^.

**Scheme 4.**
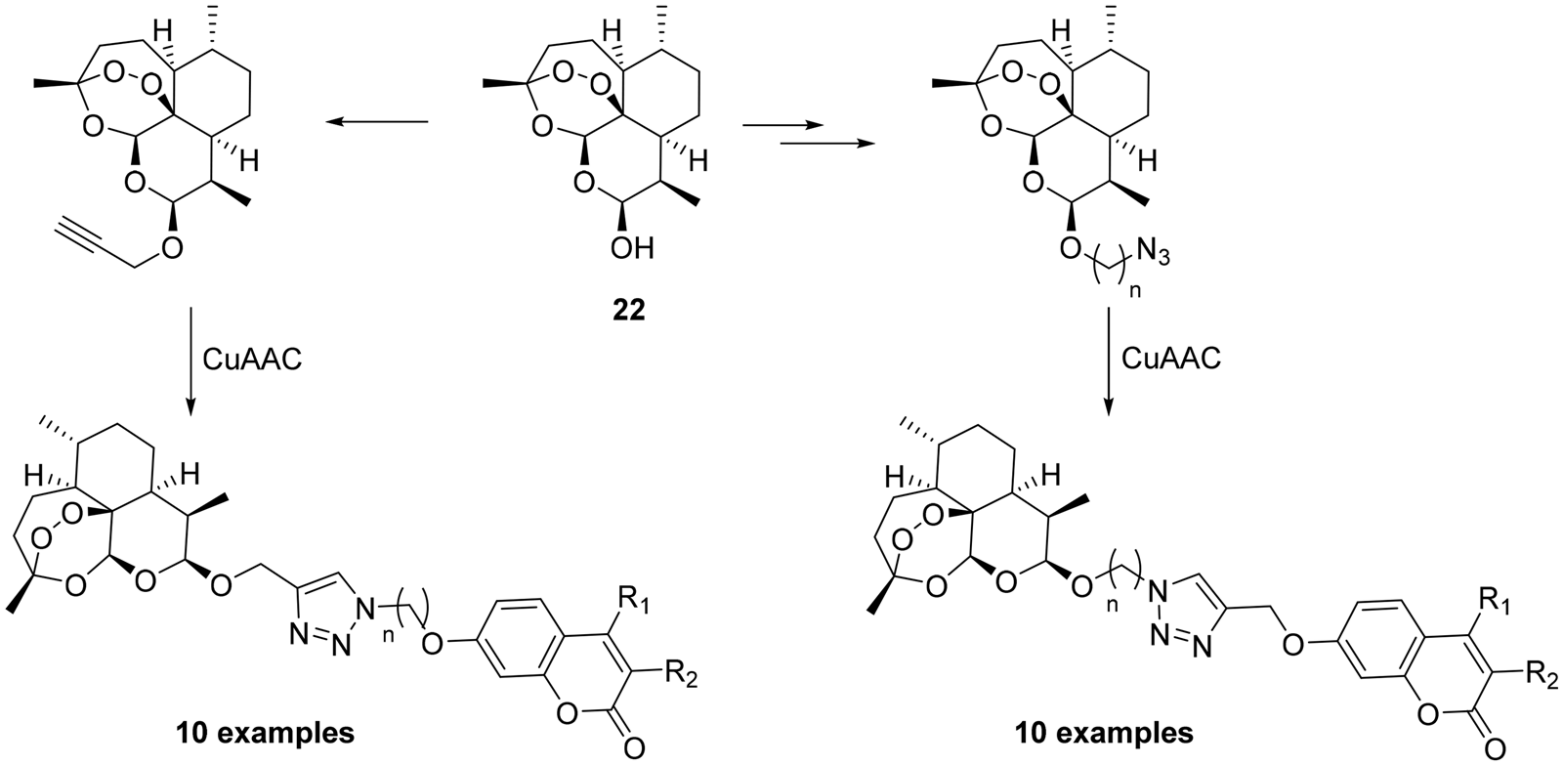
Schematic synthesis of CAI hybrids incorporating artemisin.

The fact that hCA IX overexpression has pivotal role in cancer and its expression is rather restricted to malignant cells, offers an opportunity to develop targeted therapies in the oncological field^50^. Using the principle of hybridisation, Tian et al. synthesised a series of artemisinin derivatives by means of click chemistry in which the coumarin scaffold, a selective tumour-associated CAI, was integrated^51^. As shown in Scheme 4, dihydroartemisin (**22**) has been functionalised with both the alkyne or azide group and via Huisgen’s 1,3-dipolar cycloaddition (catalysed by copper) was reacted with the coumarin counterpart.

All compounds are evaluated under normoxic and anoxic conditions against different cell lines such as MRC-5 (human foetal lung fibroblast cells, normal lung cell), HCT-116 (human colorectal cancer cell line, which does not express hCA IX in response to anoxia), MDA-MB-231 (human breast carcinoma cell line, CA IX negative), and HT-29 (human colon carcinoma cell line, overexpressing high amounts of CA IX), but without any data regarding the binding affinity against hCA IX. From the reported data, the target compounds exhibited good activity against the HT-29 cell line under hypoxic conditions displaying one- to 10-fold greater activity as under normoxic condition^51^.

**Scheme 5.**
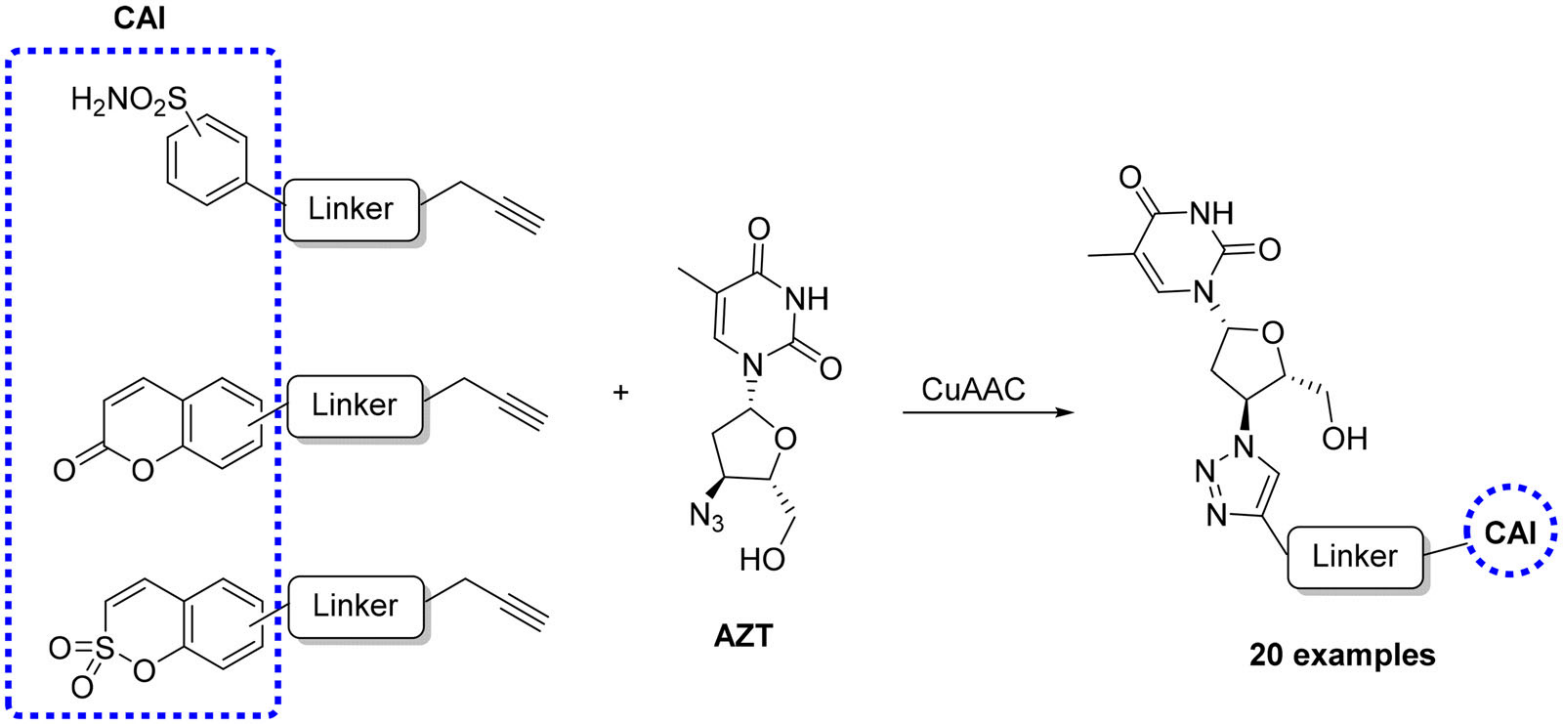
Synthesis of CAI hybrids incorporating the azidothymidine scaffold.

More recently, Berrino et al. reported CAI hybrids incorporating the azidothymidine (AZT) scaffold, a telomerase inhibitor, by using the CuAAC reaction, and obtaining a large library of derivatives bearing primary sulphonamide, coumarin and sulfocoumarin moieties (Scheme 5)^52^.

Several such compounds showed low nanomolar CA IX/XII inhibition and potent antitelomerase activity in PC-3 and HT-29 cell lines, giving the first background to develop CAI-telomerase inhibitors as promising antitumor agents.

## Click chemistry to generate other CAIs acting as antitumor agents

**Figure 7.**
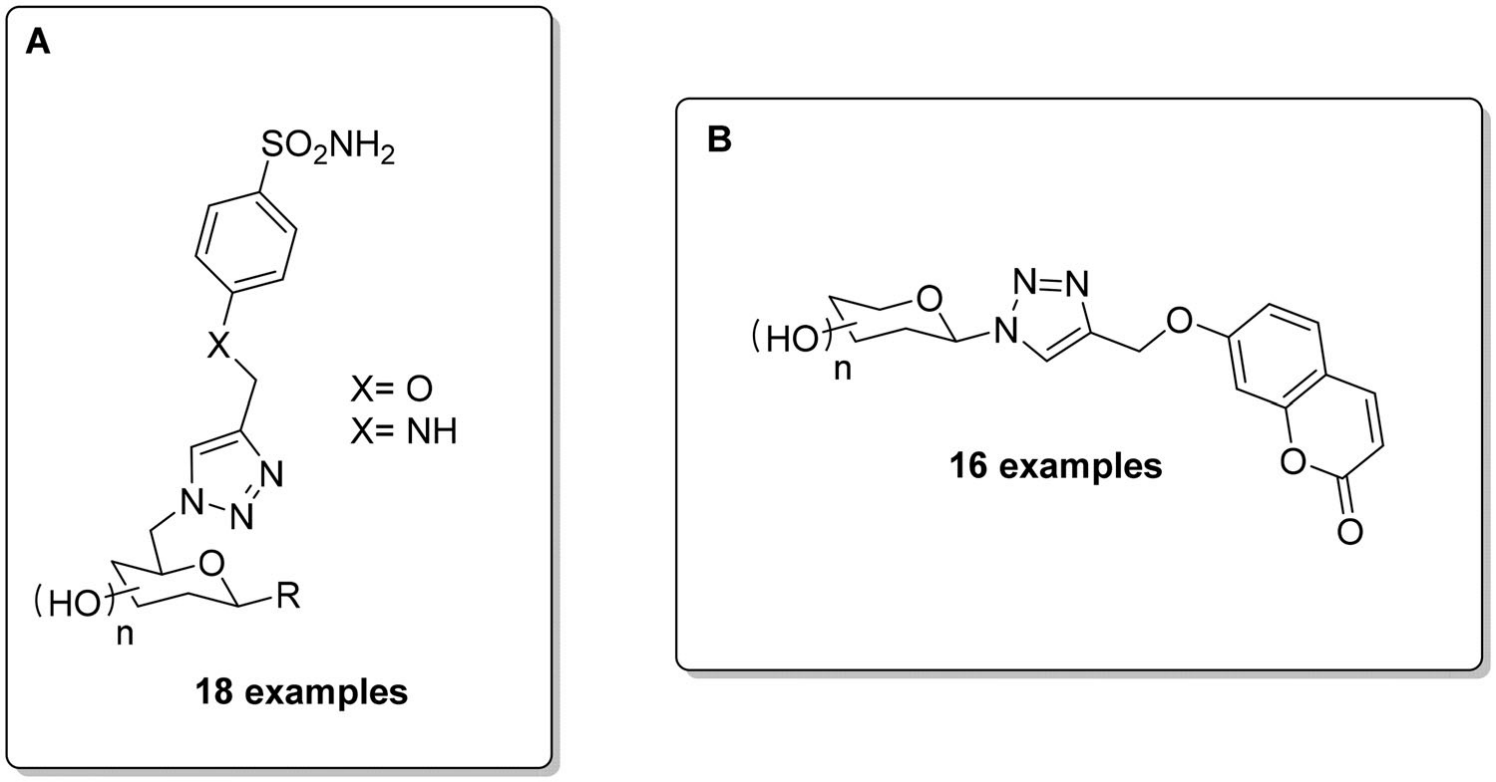
General structure of CAIs reported by Hao et al. (**A**)/Chu et al. (**B**).

As mentioned above, the opportunity for the selective targeting of the extracellular hCA IX and XII has been extensively investigated in the last two decades, also by using the click tailing approach for obtaining glycoconjugates, which due to their polar nature possess limited passive membrane permeability, enabling them to specifically bind extracellular CAs^39^^,^^40^. In this context, Hao et al. investigated the sugar-tail approach via Huisgen’s 1,3-dipolar cycloaddition, synthetising eighteen carbohydrate-based sulphonamide derivatives merging the glycosyl azide and sulphonamide-derived alkyne moiety, followed by deprotection of acetyl groups (Figure 7)^53^. Most the compounds showed high selectivity towards hCA IX and provided potential candidates to test against two cancer cell lines (HT-29 and MDA-MB-231), in which hCA IX is overexpressed. In addition, these compounds were evaluated under both normoxic and hypoxic conditions, observing an enhancement of the cytotoxicity under the last condition and, in co-administration with doxorubicin, which led to an increased cytotoxic effect.

Chu et al. applied the same sugar-tail approach by using Huisgen’s 1,3-dipolar cycloaddition with a more selective CAI chemotype, i.e. the coumarin scaffold, for targeting hCA IX and XII^54^. The activity of these compounds against three isoforms (hCA I, II and IX) was measured by using the esterase assay. The authors evaluated the potential cytotoxicity of some inhibitors on the mentioned HT-29 and MDA-MB-231 cancer cell lines, indicating that all compounds were capable of reducing tumour cell viability under normoxic and hypoxic conditions. In addition, two of the reported compounds did not show cytotoxicity towards a human normal cell line (MCF-10A) and exhibited excellent metabolic and plasma stability with half-life values ranging from 1153 min to 1843 min and with LD_50_ values >1500 mg/kg^54^.

**Scheme 6.**
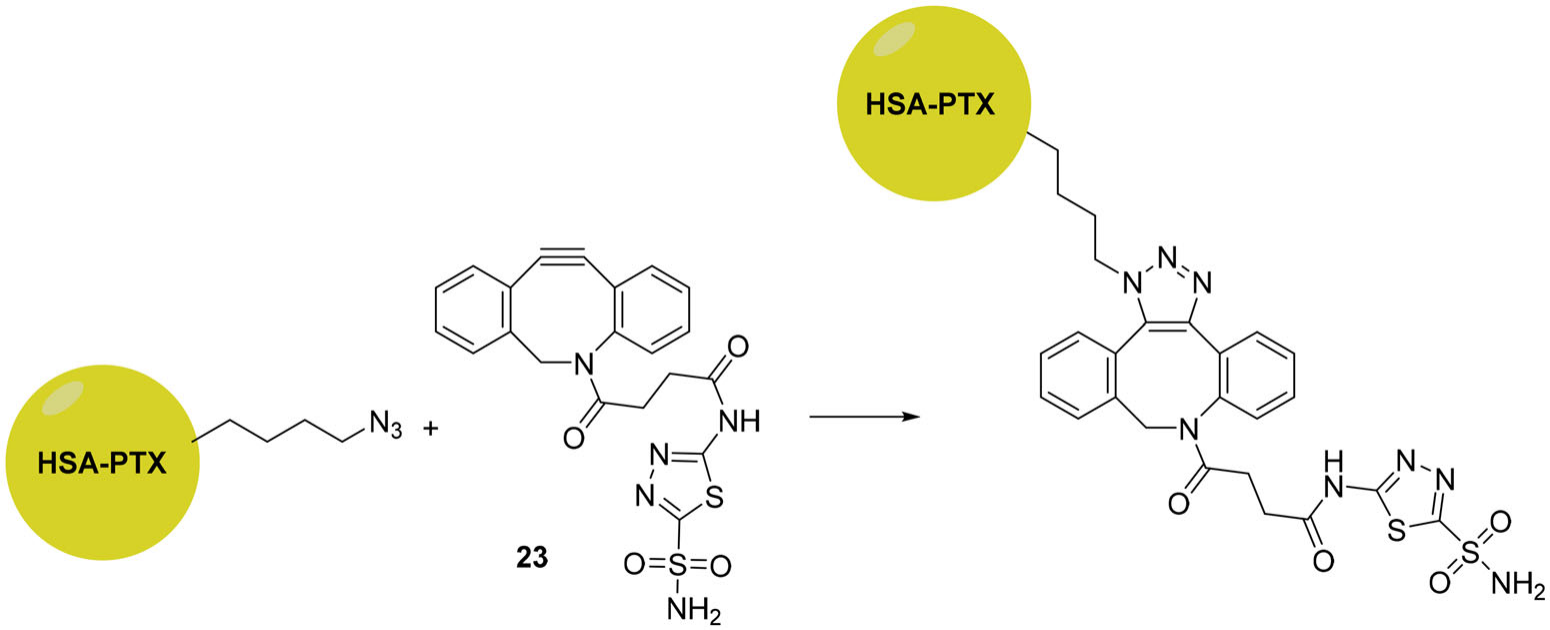
Schematic representation of the synthesis of functionalised nanoparticles reported by Tatiparti et al.

The click chemistry was also employed to develop novel delivery systems targeting hCA IX, for enhancing therapeutic efficacy of the cytotoxic agents such as paclitaxel (PTX). Tatiparti et al. used human serum albumin (HSA) as delivery system due to the fact that it presents several opportunities for covalent modification, has a low-cost, is non-immunogenic and naturally biodegradable, making it an interesting starting point for incorporating a well-known anti-cancer agent such as paclitaxel (PTX)^55^. However, to improve efficacy, the HAS nanoparticles were functionalised by using copper-free cyclic alkyne-azide click reactions, in four steps with a CAI (**23**), as shown in Scheme 6.

Subsequently, the authors carried out several studies to investigate the presence of all components used to form the delivery system such as FTIR for confirming the formation of the triazole ring, HPLC and UV spectrophotometry to check paclitaxel payload of around 11.3% w/w and the particle size. These functionalised nanoparticles wre assayed on two different cell lines overexpressing hCA IX, MDA-MB-231 and MDA-MB-468. The results showed a dose-dependent killing and also a cell viability much lower than the non-targeted preparation. In addition, hypoxic conditions increased the uptake of the formulation and enhanced efficacy in the MDA-MB-468 cell line^55^.

**Figure 8.**
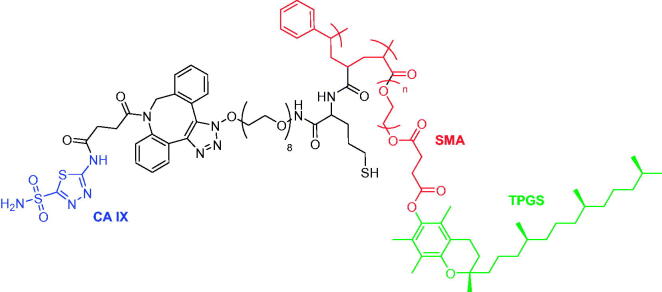
Structure of acetazolamide-oligomer (CA IX-SMA-TPGS).

hCA IX is an excellent biomarker of hypoxic tumours such as renal cell carcinoma (RCC) and, in this context, Alsaab et al. employed functionalised nanoparticles to target hCA IX in a targeted polypharmacological payload delivery against RCC cells^56^. The oligomer developed by these authors, comprised vitamin E (TPGS), a styrene maleic anhydride (SMA) ligated by click reactions with acetazolamide (ATZ), namely CA IX-SMA-TPGS and finally, encapsulated it with C4.16, a potent activators of CARP-1 (cell cycle and apoptosis-regulatory protein-1) (Figure 8). The nanoparticles were characterised by the same methods mentioned for the work of Tatiparti and were used in 3 D spheroid cells A498-RCC, a model for an *in vivo* study, in order to investigate the penetration ability of the nanoparticles in the tumour core. The obtained data showed that the hCA IX targeting oligomer efficiently reached the core of the tumour spheroid, and, when co-administered with sorafenib had a superior tumour accumulation compared to the accumulation in liver, demonstrating effective antitumor response in A498 tumours^56^.

## 6. Click chemistry in radiopharmaceutical CAIs

**Scheme 7.**
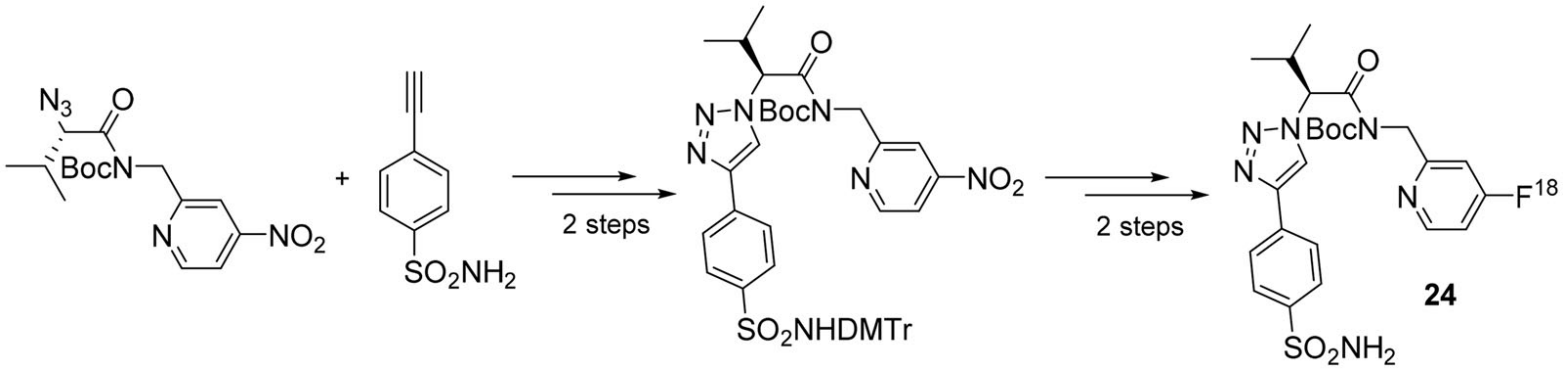
Synthesis of derivative **24.**

Click reactions, and particularly the CuAAC reactions, were used for the synthesis of several probes for obtaining radiopharmaceutical agents for nuclear imaging techniques, such as positron emission tomography (PET). Since the last review on click reactions applied to the synthesis of CAIs, published in 2010^41^, several compounds for PET have been synthesised, starting with the contribution of Mocharla et al.^57^. In a first step, the researchers synthetised a small library by target-guided *in situ* click chemistry, assembling different azides (containing fluorine fragments) with an acetylene sulphonamide (as an anchor molecule), identifying the compound with the best affinity for hCA II. The screening was performed in microtiter plates by incubating binary mixtures of anchor molecule and azides[^19^F] and hCA II in phosphate buffered saline (PBS) at 37 °C for 36 h. Among the azide moieties, the 4-fluoro-2-aminomethyl one showed the highest binding affinity with a K_d_ of 0.5 nM, and led the authors to synthetised on a preparative scale compound **24** using CuI as catalyst for the CuAAC reaction and subsequently labelled it with the isotope [^18^F] in good yields, using the 4-nitropyridine precursor (Scheme 7) accessible from the [^18^F]-fluorination techniques on deactivated aromatic rings described by Chun et al.^58^

Derivative **24** has been obtained in 15% yield with a radiochemical purity of 99.4%, as determined by radio-HPLC analysis and the time employed for the total process was 64 min, which is approximately 50% of one ^18^F half-life (t_1/2_ = 110 min), with a specific activity of the tracer from 1 to 2 Ci µmole^−1^^56^. The biodistribution profile of compound **24** showed that it was mainly accumulating in the blood, lungs, heart and kidneys with lower amounts of radioactivity founded in the stomach, muscle, brain and bone, in agreement with the known hCA II expression and distribution in these tissues^59^^,^^60^. In view of the poor blood–brain barrier (BBB) penetration observed, due to the high polarity of the triazole scaffold, compound **24** is not suitable for neurological imaging applications.

**Scheme 8.**
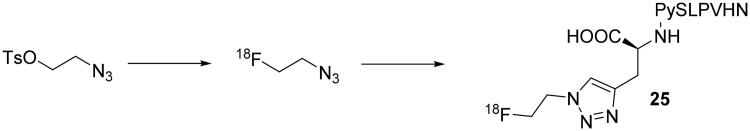
Synthesis of CA IX-P1-4–10 derivative **25.**

The employment of the radioisotope ^18^F to labelled CA inhibitors was used six years later (2019) by Jia et al. with a less investigated class of inhibitors, the peptides^61^. To date, few peptide derivatives specific for hCA IX have been developed, despite their potential advantages over antibodies for tumour imaging, such as improved tumour penetration, rapid blood clearance and less toxicity. The peptide taken into account, CA IX-P1-4–10, was previously reported by Rana et al. and has a high binding affinity and specificity for hCA IX^62^. Initially, the peptide was equipped with a terminal alkynyl moiety and, at the same time, a ^18^F-scaffold was synthetised in order to obtain, by click 1,3-dipolar cycloaddition, the labelled ^18^F-peptide **25** in an overall radiochemical yield of 35–45% and >99% radiochemical purity as reported in Scheme 8.

The novel radiochemical tracer showed good stability after 3 h at 37 °C in PBS. On the other hand, it was readily degraded in new-born calf serum (NBCS) after 3 h. PET imaging displayed clearly the tumour area with a heterogeneous intratumoral distribution and small amount of radioactivity entering also in the brain. The high expression of hCA IX induced by hypoxia in the outer core of the tumour was totally in agreement with the images reported by the authors^61^.

**Scheme 9.**
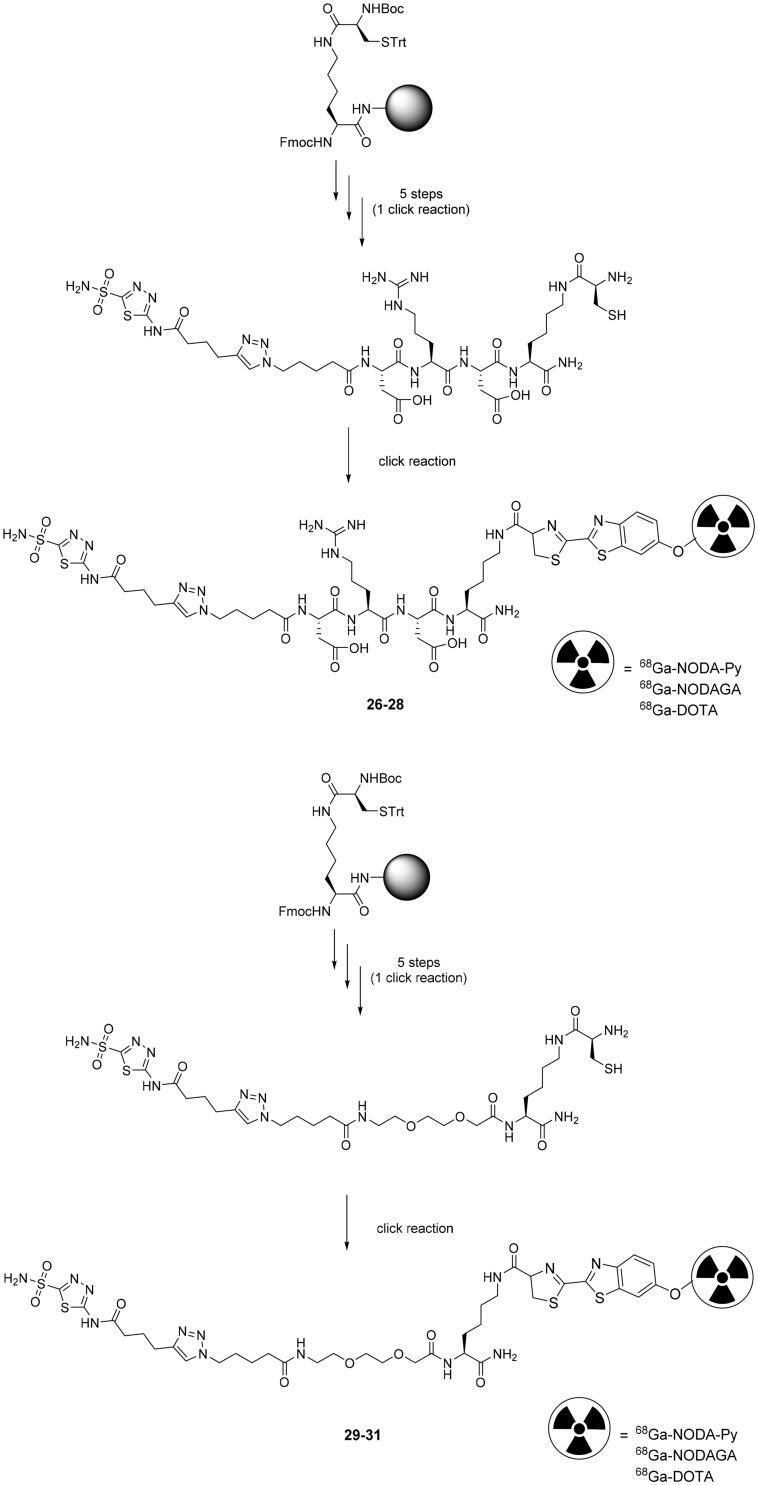
Synthesis of derivatives **26–31.** DOTA: dodecane tetraacetic acid; NODA-py: 1,4,7-triazacyclononane-1,4-diacetate pyridine

Chen et al. developed, in a two-step orthogonal labelling method, a library of six ^68^Ga-labelled acetazolamide derivatives divided in two series^63^. The synthesis has been started by solid phase synthesis by coupling in the first series a linker of Asp-Arg-Asp and in the second one a PEG2 moiety. Subsequently, the acetazolamide intermediates were incorporated to the azido-resins through a click reaction Cu(I)-catalyzed Huisgen’s cycloaddition, cleavage and global deprotection, leading to compounds **26–31** in an overall yield of 32% and 37%, respectively. At the same time, the authors prepared three cyanobenzothiazole (CBT) derivatives labelled with ^68^Ga and conjugated with acetazolamide derivatives **26–31** via an orthogonal CBT/1,2-aminothiol click reaction as highlighted in Scheme 9.

The efficiency of the click reaction was first tested at pH 7.4 demonstrating different reaction kinetics among the reported derivatives, presumably affected by the differences of chemical structures, configurations and electronic distribution^64^^,^^65^. On the other hand, at pH 9.0 in PBS the reaction was significantly improved to a yield of 85% after 30 min reaction time as opposed to 54% at pH 7.4. A nearly quantitative yield was observed after 60 min. All compounds showed excellent stability, with more than 99% of intact radioligand after 2 h incubation in PBS at 37 °C. Unfortunately, the authors did not report any biological data regarding these compounds or the binding affinity against different hCAs^63^.

## Click chemistry of fluorescent CAIs

**Scheme 10.**
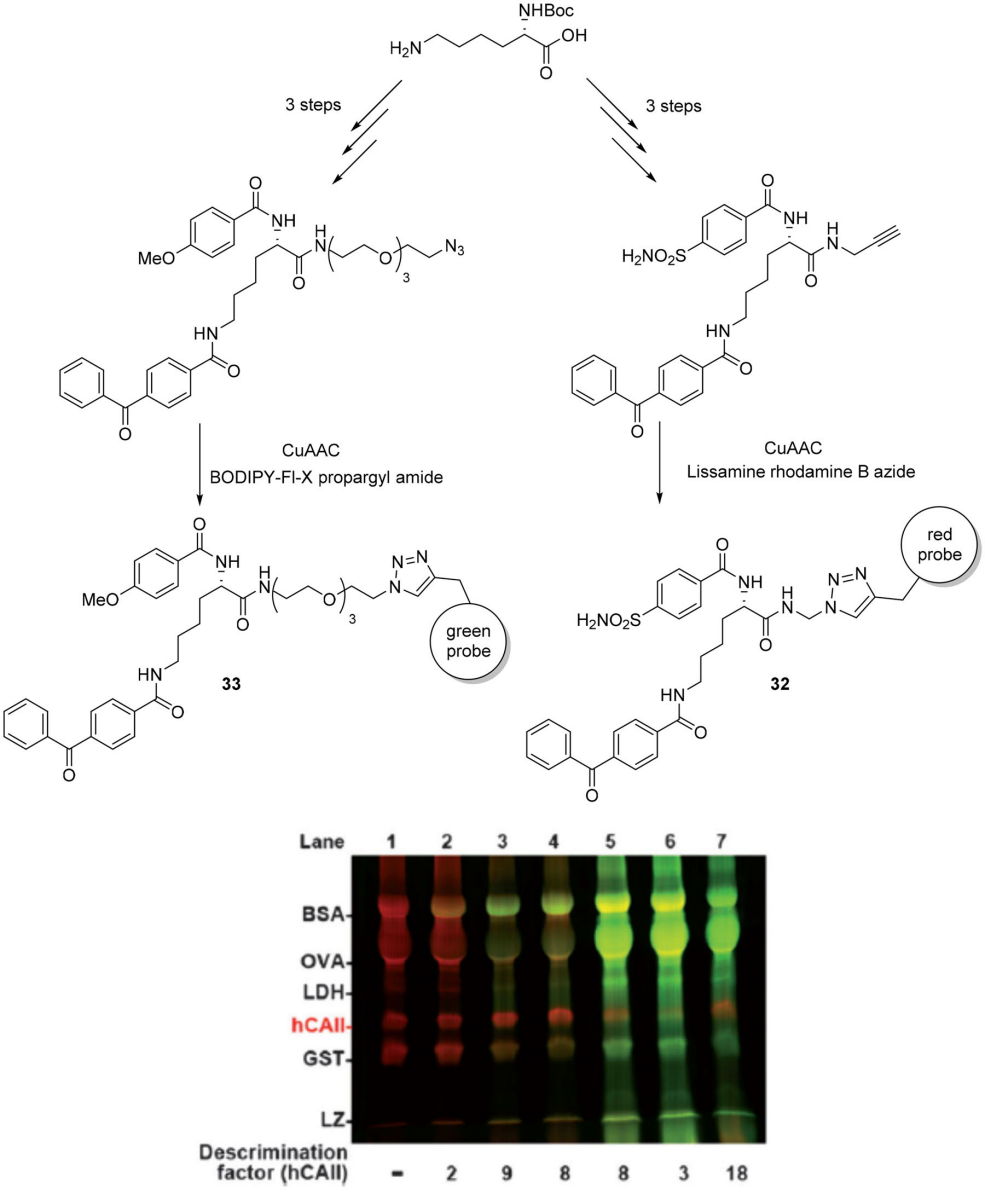
Synthesis of derivatives **32** and **33;** PAL reactions with mixture of inactive probe **33** against the active probe **32.**

The bioorthogonal click reactions are largely used over the last decade to synthesise fluorescent probes employed into the photoaffinity labelling (PAL), as a tool for target identification and to reduce the false-positive detection of abundant non-specific proteins. In this context, Sakurai et al. reported the development of a new dual-PAL strategy, where co-reaction of active/inactive probes bearing orthogonal fluorescent groups provided selective crosslinking and straightforward detection of specific-binding proteins^66^. The two fluorescent probes employed were prepared to individually detect the PAL reaction at two different wavelengths. The active probe (**32**) yields a red fluorescent bands, on the other hand, the non-specific probe (**33**) predominantly react with off-target proteins to give green bands (Scheme 10). This concomitantly employment of two probes could be useful to reduce the false-positive reaction products and the less efficient binding of the active probe to non-specific proteins. The PAL model reported by these authors is designed to target selectively hCA II among an excess amounts of an inactive analogue (100- to 1000-fold excess) reaching the detection of hCA II at 1% (w/w) abundance in cell lysate. In contrast, when the same active probe (**32**) was used by itself, hCA II could be detected only at a higher abundance (>5% w/w).

The fluorescent moiety could preinstall as a part of the probe structure or introduced by an excellent alkyne/azide-reactive fluorescent reaction through click chemistry after the PAL reaction. However, high concentration (>100 µM) of inactive probe is needed to block non-specific proteins and a ratio of 5:1 with active probe is needed to show the best activity with a discrimination factors greater than 10^66^. Subsequently, the authors employed this system in a mixture of five non-specific proteins (bovine serum albumin, BSA; ovalbumin, OVA; lactate dehydrogenase, LDH; glutathione S-transferase, GST; and lysozyme, LZ) containing 1% (w/w) of hCAII. Immediately, hCA II is detected as a red band (specific-binding protein) among the other proteins detected as predominantly green bands (non-specific proteins). In addition, was reported the employment of this system in a more biologically relevant context as HeLa cell lysate containing 0.1–10% hCAII, showed the active probe-hCAII cross-linked product as a singly distinct red band in the presence of a multitude of other proteins as smeared green bands. Finally, the authors reported the products separation in 2D increasing the resolution problems of overlapped bands present in 1D analysis.

**Scheme 11.**
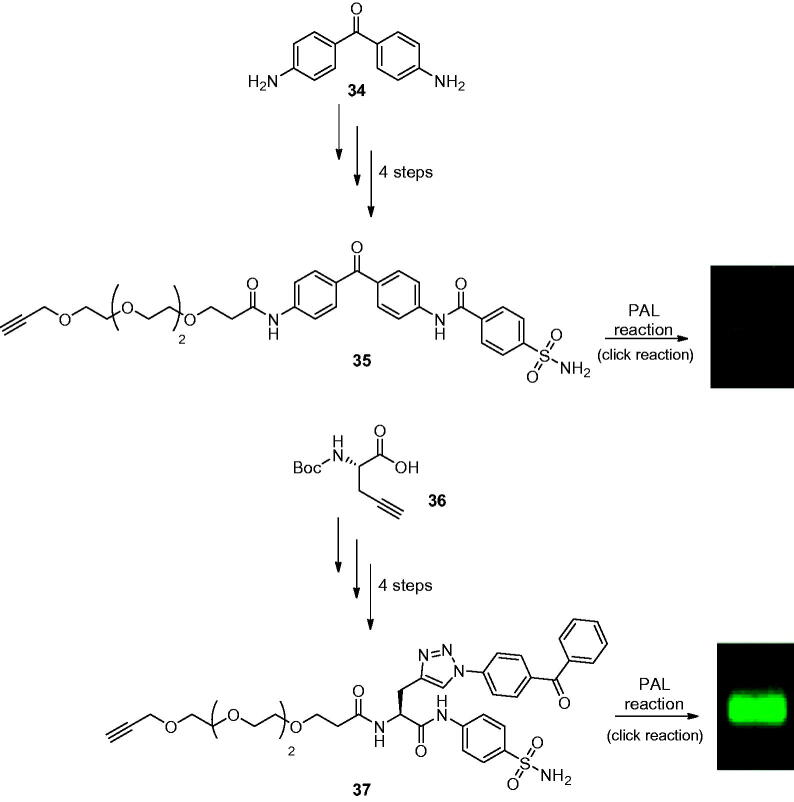
Synthesis of linear PAL probes **35** and of the branched PAL probes **37** labelling hCA II with fluorescence band.

The development of PAL probes targeting CAs were investigated by Poulsen’s group in 2017, by incorporating a wide selection of primary sulphonamides possessing different structural features, a linker group as well as linear and branched arrangements of the probe components for selective detection of hCA II^67^. The design of these molecules has been done by taking into account hydrophilic linkers such as ethylene glycol to counterbalance the hydrophobicity of the benzophenone moiety (UV reactive moiety) and its scarce water solubility. The two different classes of designed probes, the linear (**35**) or branched (**37**) one, were synthesised in two different ways. The linear PAL probes were synthesised through sequential amide coupling reactions starting from the 4,4′-diaminobenzophenone scaffold (**34**). On the other hand, the branched PAL probes were synthesised from a Boc-L-propargylglycine (**36**) with a panel of different amino benzenesulfonamides as outlined in Scheme 11.

As in the experiments to Sakurai’s group, previously discussed, selectivity towards the labelling of hCA II in the presence of excess non-specific proteins (such as BSA, OVA) in a total concentration of 4.3%, w/w of hCA II has been observed. From the general point of view, all linear probes showed strong non-specific labelling of BSA and weak non-specific labelling of OVA demonstrating that linear structures have a greater tendency to undergo non-specific labelling than probes with a branched structure, attributed to the relative flexibility of the former probe design^68^. Indeed, the authors reported the selectivity index (SI) for each probe over the non-specific proteins, showing for probe **35** a SI for hCA II of 2.58 fold over BSA and 22.0 fold over OVA. Unfortunately, it resulted only in a faint hCA II band in all PAL experiments. However, the most favourable hCA II selectivity was achieved with the branched probe **37** with a SI of 20.0 fold against BSA and 33.8 fold over OVA and showed a strong hCA II-selective band. Interestingly, the results reported in the PAL experiments did not directly correlate with the affinity for hCA II as shown by the IC_50_ values reported in the work, as derivative **37** has a moderate affinity for hCA II. For this reason, the authors investigated the PAL probe–protein binding, protein crosslinking efficiency, and CuAAC efficiency by native state mass spectrometry and in-gel fluorescence showing for all probes a moderate binding formation with hCA II as in the case of complex [hCAII:**37**] where a substantial portion of **37** binding to hCA II active site but not crosslinking with CA II on exposure to UV, thus suggesting that the probe architecture might significantly affect the binding and crosslinking efficiency to the hCA II active site. Additionally, correlation of in-gel fluorescence and the crosslinking efficiency determined by MS analysis was not straightforward, as probe **35** generated only a faint fluorescence despite showing the highest crosslinking yield, whereas the branched probe **37**, which showed >4.5-fold lower crosslinking yields than probe **35**, gave moderate fluorescence intensity, probably due to the different accessibility to the biorthogonal reaction in the complex protein-bound probe. Thus, the conformational flexibility of the probe has been suggested to be a critical feature that can impact both target-selective binding as well as non-specific labelling, crosslinking efficiency and CuAAC reaction efficiency.

**Scheme 12.**
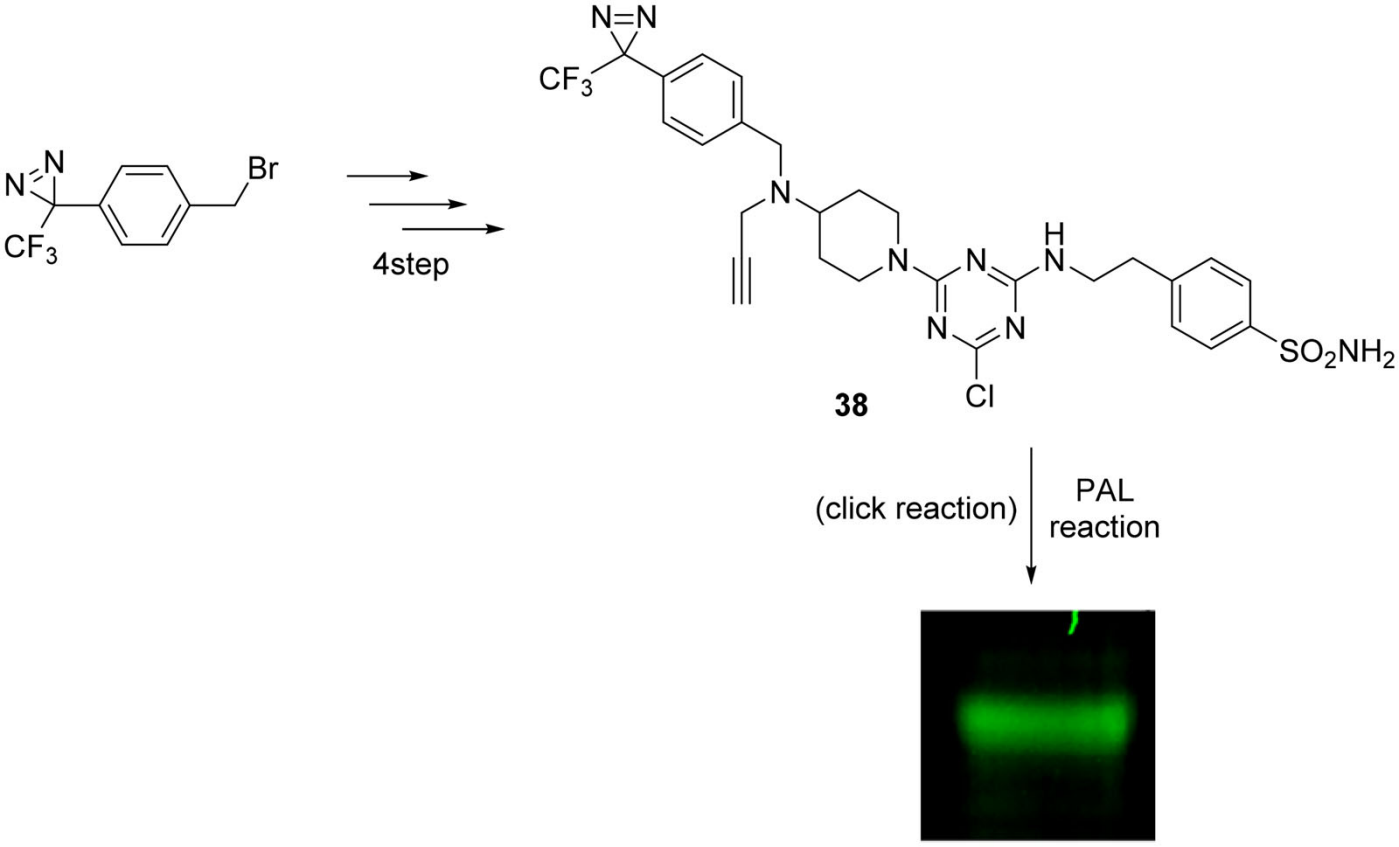
Synthesis of compound **38** and photoaffinity labelling of recombinant CA IX.

Zhao and Burgess in 2018 synthesised trough CuAAC reaction, a CAI for targeting the tumour-associated isoform IX for PAL experiments, as reported in Scheme 12^69^. As starting point, the authors conjugated derivative **38** with recombinant CA IX in aqueous buffer at pH 7.4 and illuminated it at 365 nm, observing a good fluorescent band at 55 kDa, fully compatible with recombinant CA IX. The next step was the employment of compound **38** in a more complex system as “Triple negative” breast cancer cells, MDA-MB-231, since are well-known to overexpress CA IX^70^ revealing the presence of hCA IX in the cell lysate before pull-down.

In the last years, Sakurai groups continued to study the employment of CAI in PAL with gold-nanoparticle-based probes to enhance protein binding and labelling efficiency^71^. The gold nanoparticles give an interesting opportunity to load and choose the ratio of photoreactive group, as well as the clickable functionality by click chemistry immediately prior to PAL. The authors immobilised all loaded functionalities via the dithiol moiety of lipoic acid with a diazirine group as a small photoreactive group, a precursor azide group and a PEG moiety as a hydrophilic spacer which improves the colloidal stability. Indeed, the surface azide group tends to agglomerate the gold-nanoparticles in aqueous buffer due to the neutral charge and hydrophobic property of azides. The authors employment this platform to target bovine CA II (bCA II) by CuACC with alkyne benzenesulfonamide and verified the correct formation of the 1,2,3-triazoles scaffold by MALDI-TOF MS. At this time, the nanoparticles were tested at 20 nM in the presence of excess amounts of a non-specific protein, BSA showing a photo-crosslinked of bCA II with high selectively over BSA. On the other hand, when the authors tried to detect hCA II in the HL60 cell lysate, they could not visibly detect the corresponding protein band, which may be attributable to the low labelling yield and the low abundance of hCA II in HL60 cell lysate^72^.

**Scheme 13.**
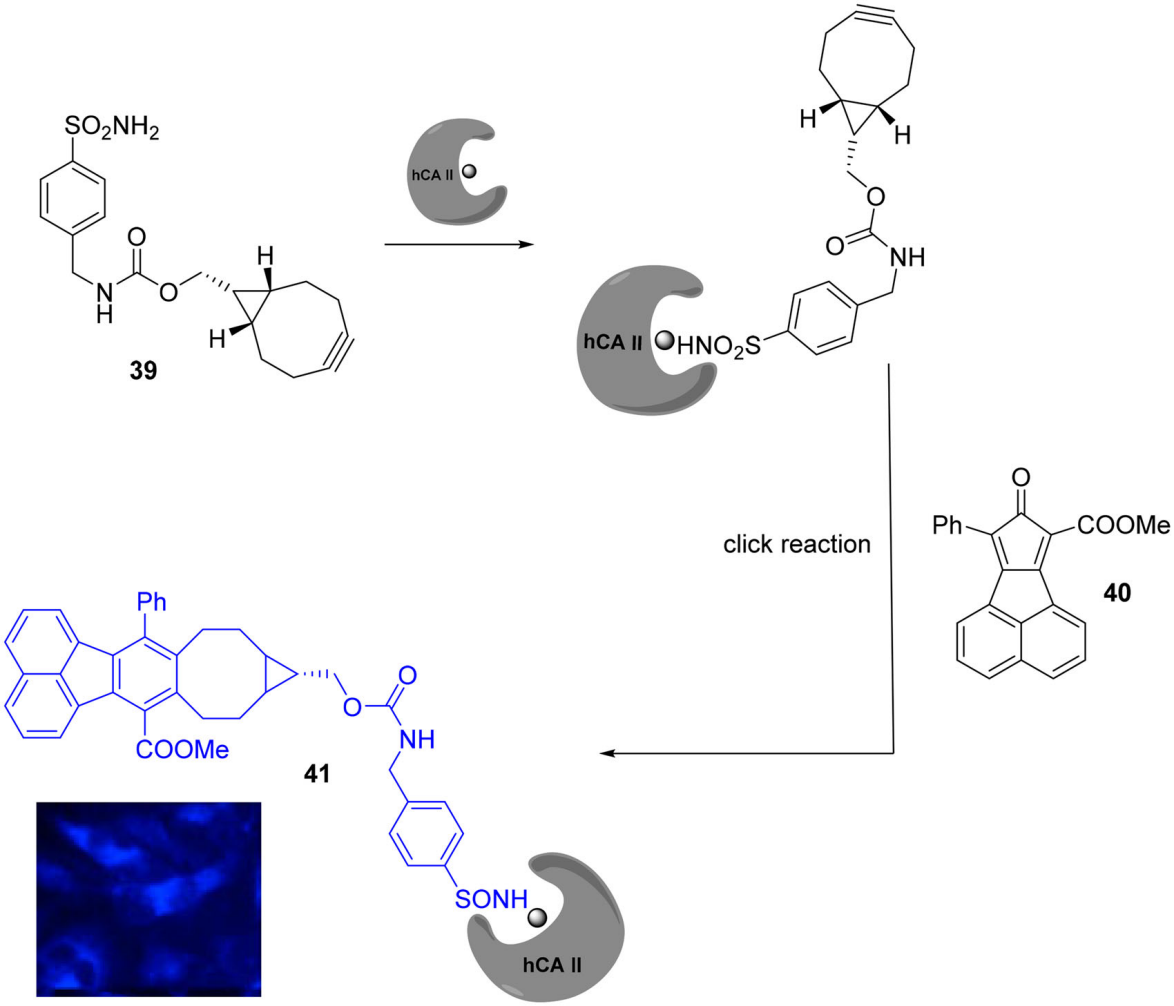
Development of bioorthogonally activated smart probe **41.** The blue fluorescence represents DAPI (4',6-diamidino-2-phenylindole) channel.

Bioorthogonal chemistry was not exclusively employed in PAL technology for the synthesis of fluorescent CAIs. Ji et al reported the development of bioorthogonally activated smart probes, where the florescence is “turned on” upon the click reaction between the two used reagents, thus making the tedious washing steps involved in biomolecules tagging unnecessary^72^. The high fluorescence turn-on ratio of fluorophore probe (**41**) was achieved by one step straightforward click reaction among a strained alkyne bearing a sulphonamide moiety (**39**) with a cyclopentadienone derivative (**40**) without any catalyst as highlighted in Scheme 13.

This smart probe was employed to selective label in two step the hCA II isoform in Hela cells showed strong blue fluorescence. In addition, the authors to confirm that the blue florescence was mainly the result of the smart probe bounded to hCA II, the Hela cells are pre-treated with a non-fluorescence CAI followed by the incubation with the probe showing a significantly decrement of blue fluorescence intensity compared to the cell treated with only the fluorescent probe confirming the selectivity of the target^72^.

**Figure 9.**
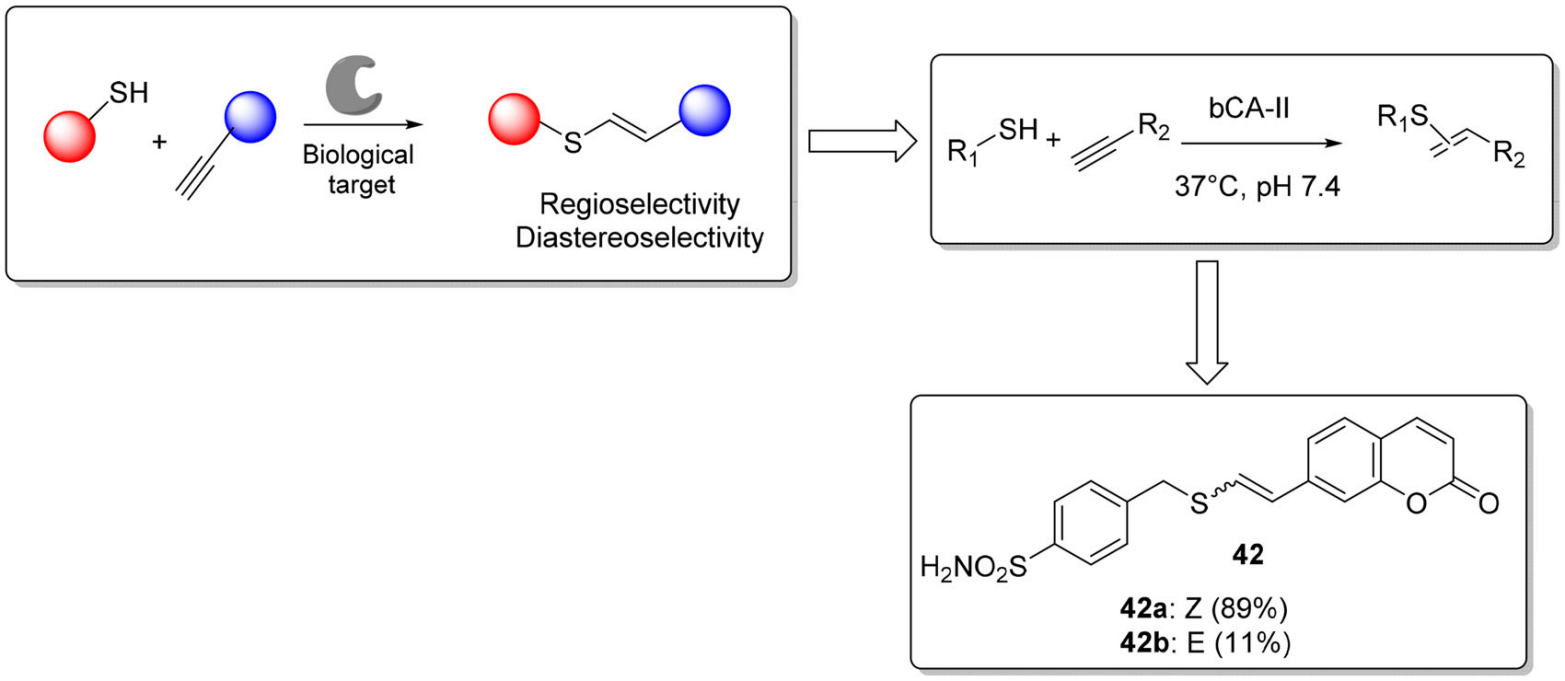
Thiol-yne reaction and structure of the hit compound **42**.

Lossouarn et al. used the thiol-yne click chemistry as a new ligation tool for the protein-directed synthesis of ligands and their detection^73^. Again the authors chosen the bovine CA II (bCA II) isoform as model protein to evaluate the potential of thiol-yne reaction and employed a thiol sulphonamide derivative as anchor for bCA II, incubated it with a series of alkynes derivatives in order to drive the synthesis of the most potent ligands. They found a ∼2- to 10-fold acceleration (depending on the protein batch) of the thiol-yne addition reaction leading to the vinyl sulphides **42** and driving the formation of a prevalent diasteoisomer compared to the other one (Z/E: 89:11) (Figure 9), which was confirmed also by different experiments. Thus, the reaction occurred in the active site of bCA II and not in another hidden pocket or binding site.

The biological activity of two diastereoisomers (**42a** and **42 b**) showed a different affinity, confirming that product **42a** is the more potent inhibitor of bCA II (0.33 µM) and the possibility of using thiol-yne click chemistry as protein-directed synthesis of ligands. Finally, for compound **42** it was observed an enhancement of fluorescence only with bCA II indicating the probe specificity for bCA-II binding site^73^.

**Figure 10.**
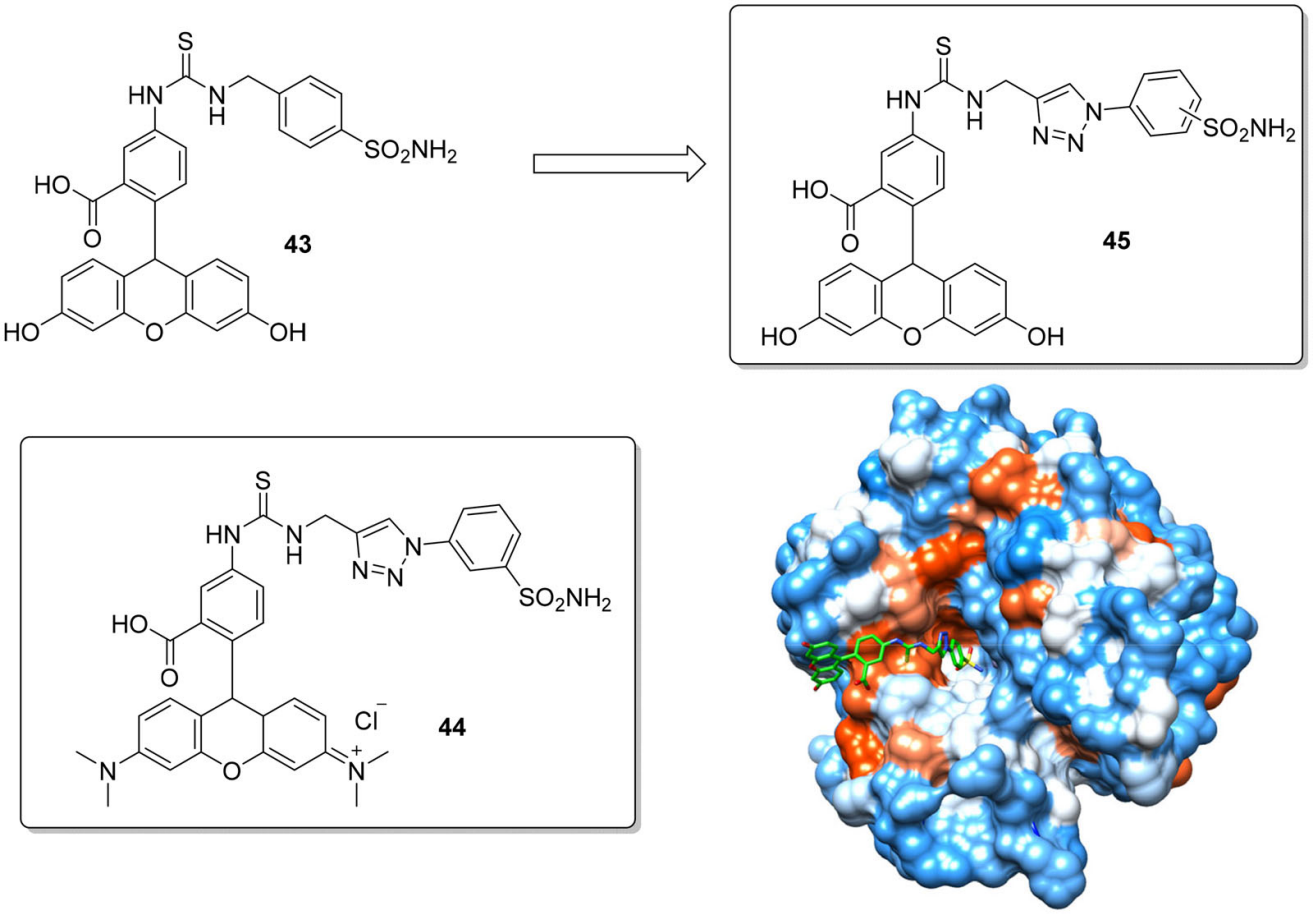
Structure of fluorescent CAIs **43–45** and crystallographic structure of **45** with hCA II.

Fluorescent sulphonamides are employed in the last decades also as essential probes for demonstrating their role in different diseases. In particular, hCA IX was extensively studied to deeply understand the role played in acidification of the tumour microenvironment^74^. Carta et al. reported two novel fluorescent CAIs to overcome the relatively low water solubility of previously reported such fluorescent probes which limited their use in biological experiments^75^. The used synthetic approach was the replacement of alkyl spacers with a 1,2,3-triazolyl ring by the click chemistry methodology, which due to the rich presence in heteroatoms should increase water solubility (Figure 10). In addition, the fluorescent probe was replaced with a (6-dimethylamino-xanthen-3-ylidene)-dimethyl-ammonium chloride scaffold for compound **44** in order to increase the water solubility as well as the membrane impermeability compared to the structurally-related derivative **43,** again for obtaining a selective inhibitor of the membrane-bound isoforms over the cytosolic ones.

The replacement of the methyl spacer from (**43**) with a 1,2,3-triazolyl moiety (**45**) showed a 3-fold increase of the phosphate buffer (pH 7.4) solubility (from 1.2 mg/mL to 3.8 mg/mL) and for this reason derivative **45** was crystallised complexed with hCA II. It showed for the tail portion a very different orientation compared to structurally similar compounds devoid of the triazole moiety reported by Alterio et al. (Figure 10)^76^. Finally, the novel fluorescent probes were assayed against five human isoforms (i.e. hCA I, II, IX, XII, XIV) and showed higher affinity for the transmembrane isoforms compared to the parent compound **43.**

## Click chemistry on multivalent CAIs

**Figure 11.**
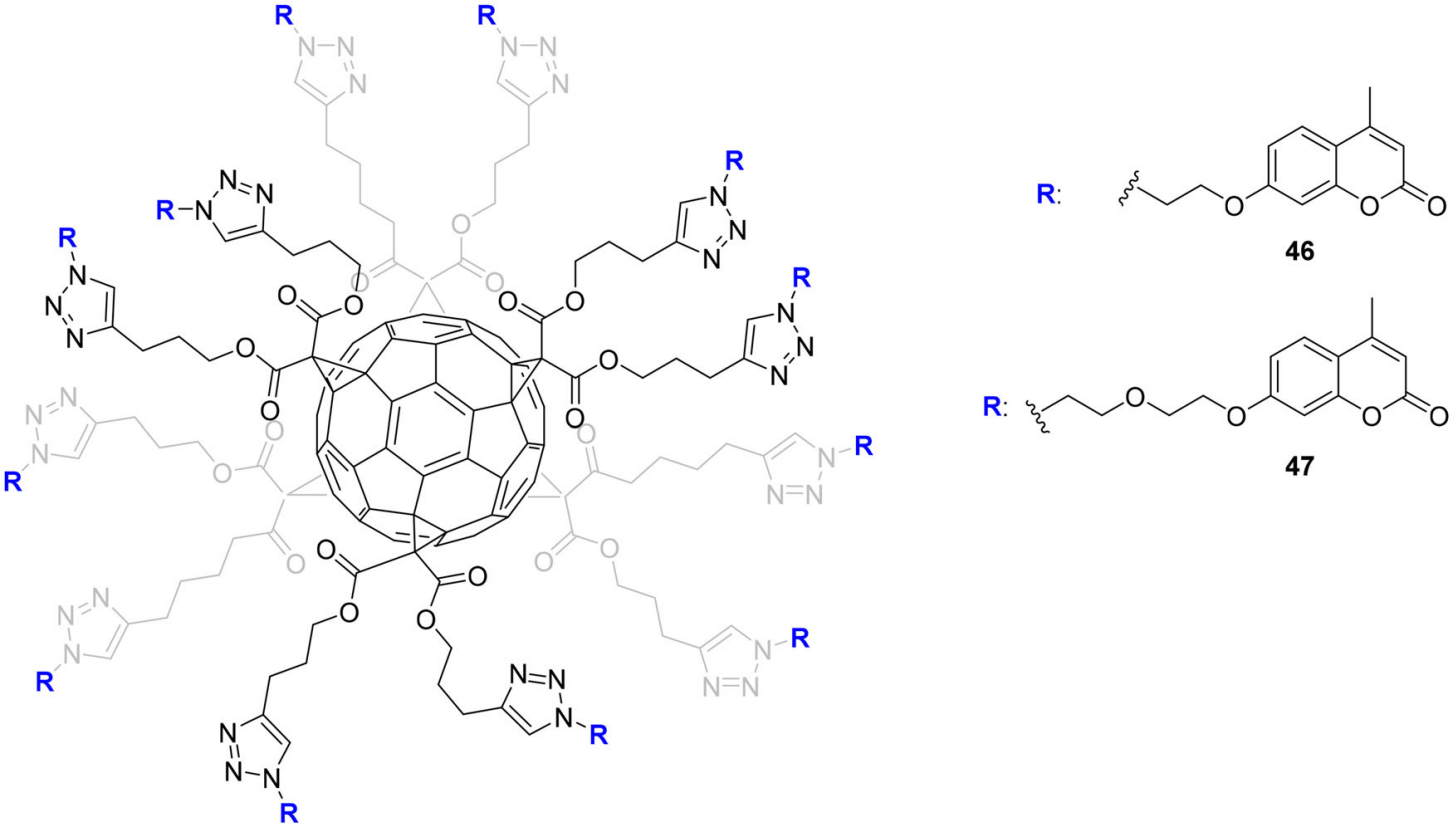
Multivalent coumarin derivatives **46** and **47**.

The multivalent approach in the design of novel CAIs was successfully applied with a variety of inhibitors such as sulphonamides, dithiocarbamates, and coumarins^30^^,^^77^. This interesting strategy took into account that thermodynamic modifications in binding energy when switching from the monovalent to the multivalent systems can be exploited to improve affinity and selectivity. This means that an inhibitor may become more effective and more selective in a cluster than alone. Furthermore, the multivalency strategy could be employed to transform low and weakly selective inhibitors into potent and selective ones^78^. However, multivalent enzymatic inhibition is not particularly easy to achieve. In fact, the presence of multiple inhibitors on a molecular platform did not necessarily lead to an effective multivalent system since different parameters need to be considered for obtaining an effective platform, such as topology, structure, and valency of the platform; length, and rigidity of the ligation system, etc.^78^. Multivalent nanoconstructs are generally prepared by the conjugation of inhibitors onto multifunctional (bio)molecular chemical frameworks using different conjugation methodologies, among which also the click reactions^78^. Since the fullerene scaffold is an excellent multivalent platform with exclusive physical, electrochemical, and chemical properties, Vincent’s and Supuran’s groups decided in 2015 to explore, via click-type CuAAC reaction, the possibility to install different coumarin moieties on it, offering thus the possibility of designing selective inhibitors^79^. The two fullerenes C_60_ **46** and C_60_ **47** bear twelve coumarins differing only in the length of the spacer group linking the ligands and the fullerene core as depicted in Figure 11.

**Figure 12.**
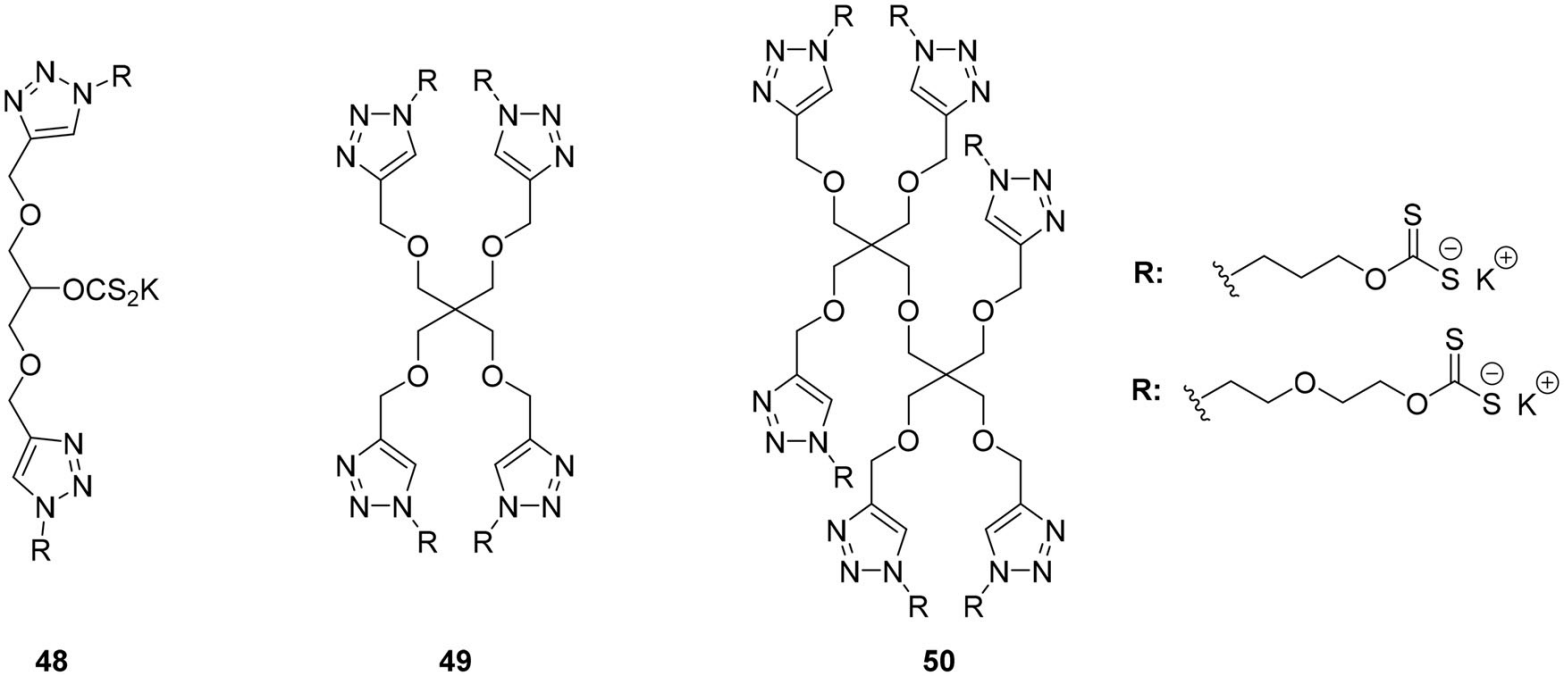
Multimeric xanthate systems **48–50**.

Multivalent systems showed in each case, better inhibitors than monovalent inhibitors. In addition, the multivalent systems affect the selectivity against the different isoforms showing weak inhibition against the cytosolic isoform I and II compared to the monovalent inhiibtors^79^. The same research groups investigated multivalent polyol-scaffolds decorated with xanthate derivatives as CAIs of types **48–50** (Figure 12)^80^.

Xanthates are well known to be not very stable compounds, as they are sensitive to acidic pH, high temperature, humidity and time, being hydrolysed to the starting alcohol and CS_2_ as products^81^. The platform synthesised by these research groups, containing three (**48**), four (**49**), and six (**50**) xanthate moieties mainly showed inhibitory activity against hCA I. Also in this case, the platform showed significantly stronger inhibition compatred to the monomeric species, but no strong multivalent effect was observed^80^.

**Figure 13.**
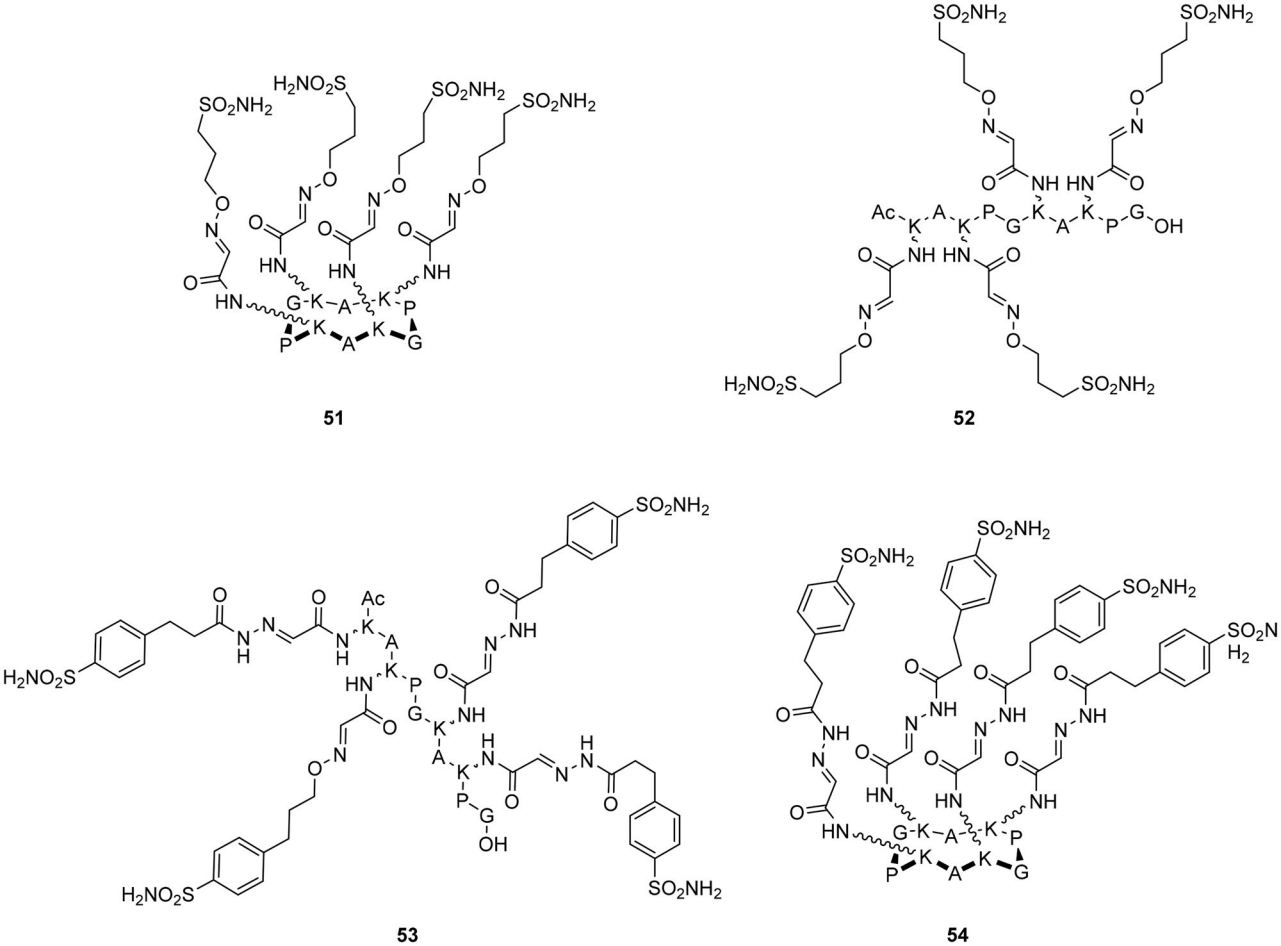
Multivalent CA inhibitors **51**–**54** from functionalised peptide scaffolds.

Winum’s group, in 2017, synthesised cyclic (**51**, **54**) and linear (**52**, **53**) peptides conjugated with multiple sulphonamide moieties under mild conditions and in high yields using metal-free click-type bioconjugation methodologies such as hydrazone and oxime ligations, as depicted in Figure 13^82^.

From the inhiibtion assay, it has been observed that the multivalent oxime conjugates **51** and **52** were less potent than the monovalent system against the membrane-bound isoforms hCA IX and hCA XII. The only improvement in potency was observed against hCA II. On the other hand, multivalent hydrazide conjugates **53** and **54** showed an improved potency against hCA II, IV, and XII compared to the monovalent inhibitors. Finally, the cyclic products **51** and **54** displayed a better inhibition activity than the linear ones (**52**, **53**)^82^.

## Conclusions and future prospects

This review describes the last 10 years of click chemistry as a useful and versatile synthetic tool, widely applicable for the development of novel CAIs, as well as their pharmacological applications. Click reactions are easy to perform under ambient conditions, give high reaction yields, have a good regiospecificity, and are tolerant to standard biological conditions, identifying the role of 1,3-dipolar click cycloaddition of alkynes and azides as the gold standard reaction to generate compound libraries and accelerating the process of finding and optimising lead candidates in all CAI classes. These reactions led many research groups to develop potent CAIs and afforded the possibility to modulate their selectivity towards various isoforms of pharmacological interest. However, the introduction of copper species into biosystems and living organisms raises the issue of potential toxicity, and thus, in the last years, the strain-promoted cycloadditions of cyclic alkenes and alkynes has established themselves as powerful alternative tools in bioconjugations. This review thus provides a general overview on the diversity of click chemistry developments in the CA field, which provided an important chemical strategy useful for different applications involving CA modulators. However as seen in the article, the click chemistry was mainly employed to develop CA IX/XII inhibitors, useful as anticancer agents and diagnostic tools^14^^,^^74–76^^,^^83^. Indeed, the recent validation of these two targets in the oncological field^77^^a,^^84^^,^^85^, led to a very large number of studies for designing potent/selective inhibitors, and many of them made use of click chemistry reactions. On the other hand, the CAIs possess other relevant therapeutic uses, which were not considered so far from the click chemistry viewpoint. For example, there are several antiobesity agents, such as topiramate and zonisamide, which inhibit the mitochondrial isoforms CA VA and VB, and induce a potent inhibition of lipogenesis^86^. At this moment, no click chemistry studies have been reported for the design of novel antiobesity such agents, as well as for diuretics^87^ antiepileptics^88^ anti-neuropathic pain or other agents useful for the treatment of neurologic disorders^15^^,^^89^, which target various other CA isoforms than CA IX and XII.

The antiinfectives based on CAIs started to be seriously considered only in the last decade^90^, and several highly interesting studies for the design of antibacterials^91–93^ and antifungals^94–96^ which target CAs present in pathogenic organism have emerged. However, in these cases the use of click chemistry has not yet been contemplated, as it is also the situation for CA activators^97^, which might be useful for the management of emotional/fear memory therapy and several cognitive disorders^98^^,^^99^. As outlined here, there are many other hot topics in the CA research field in which click chemistry reactions may contribute to the development of highly interesting inhibitors and activators, and hopefully such studies will be stimulated to be performed by this review.
